# Influenza A virus infection induces immune dysregulation in the placenta and fetus without vertical transmission in nonhuman primates

**DOI:** 10.1126/sciadv.aee9861

**Published:** 2026-10-09

**Authors:** Orlando Cervantes, Sidney Sun, Hazel Huang, Briana Del Rosario, Hong Zhao, Amanda Li, Andrew Vo, Gygeria Manuel, Roslyn Van Abel, Jeff Munson, John Cornelius, Raj P. Kapur, Miranda Li, Edmunda Li, Hanning Li, Alexandra Christodoulou, Melissa Berg, Britni Curtis, Elizabeth Miller, Shannon Ruff, Brenna Menz, Solomon Wangari, Audrey Germond, Audrey Baldessari, Chris English, W. McIntyre Durning, Thomas B. Lewis, Megan N. Fredericks, Mara Maughan, Austyn Orvis, Michelle Coleman, Lakshmi Rajagopal, Deborah H. Fuller, Kristina M. Adams Waldorf

**Affiliations:** ^1^ Department of Global Health, University of Washington, Seattle, WA, USA.; ^2^ Department of Obstetrics and Gynecology, University of Washington, Seattle, WA, USA.; ^3^ Case Western Reserve School of Medicine, Cleveland, OH, USA.; ^4^ School of Medicine, University of Washington, Seattle, WA, USA.; ^5^ Department of Psychiatry, University of Washington, Seattle, WA, USA.; ^6^ Department of Lab Medicine and Pathology, University of Washington, Seattle, WA, USA.; ^7^ Department of Pathology, Seattle Children’s Hospital, Seattle, WA, USA.; ^8^ Washington National Biomedical Research Center, Seattle, WA, USA.; ^9^ Department of Microbiology, University of Washington, Seattle, WA, USA.; ^10^ Oregon Health & Science University, Portland, OR, USA.; ^11^ Center for Global Infectious Disease Research, Seattle Children’s Research Institute, Seattle, WA, USA.; ^12^ Department of Pediatrics, University of Washington, Seattle, WA.

## Abstract

Influenza A virus (IAV) infection during pregnancy is associated with stillbirth and preterm birth. We hypothesized that maternal IAV infection disrupts placental and fetal immune networks in ways that correlate with disease severity. Pregnant pigtail macaques were inoculated with IAV [A/California/07/2009 (H1N1)] and underwent delivery and fetal necropsy 5 days postinfection (*N* = 11), with comparison to uninfected controls (*N* = 16). Stillbirth occurred in 18% of infected pregnancies and in no controls. Although vertical transmission was not observed, low levels of viral RNA were detected in two placentas. Maternal infection was associated with induction of a type I interferon response in the placenta and altered innate and adaptive immune cell populations in the fetus. Maternal disease metrics (e.g., lung viral load and interferon-α) were rarely associated with placental immune perturbations but frequently linked to fetal immune cell populations (CD4^+^ and CD8^+^ T cells and monocyte subsets). Collectively, these data indicate that maternal IAV disease severity is associated with dysregulated fetal immunity.

## INTRODUCTION

Influenza A virus (IAV) infections remain a global public health threat, which is especially pronounced in immunologically vulnerable populations such as pregnant women (*1*, *2*). IAV is a negative-sense, single-stranded RNA virus composed of eight segments. Pandemics occur when reassortment of viral segments produces new combinations of its surface glycoproteins, hemagglutinin (HA) and neuraminidase. Epidemiologic evidence from several IAV pandemics (e.g., 1918, 1957, 1968, and 2009) indicates that IAV infections in pregnancy impart a greater risk of maternal mortality and adverse pregnancy outcomes, such as stillbirth (*3*–*14*). There may also be a long-term risk of neuropsychiatric disease in the children born to pregnant women infected with IAV or other infectious diseases, which has been reported in multiple populations (*15*–*22*).

The effects of placental and fetal exposure to maternal IAV have been studied primarily in murine models. Experimental IAV infection in pregnant mice and rats (e.g., H1N1 and H3N2) has been associated with a range of adverse outcomes, including preterm birth, low birth weight, and stillbirth (resorptions) (*23*–*27*). These models demonstrate robust activation of innate and adaptive immunity in the maternal compartment, with increased monocytes, neutrophils, and T cells detected in the lung, placenta, and gut-associated lymphoid tissue. In mice, IAV can disrupt progesterone and prostaglandin signaling in the placenta, which was associated with adverse perinatal outcomes and placental remodeling (*26*, *28*). Severe maternal IAV-associated vascular inflammation has been associated with placental growth restriction and fetal brain hypoxia (*23*). In contrast, relatively few studies have examined fetal immune perturbations following in utero exposure to maternal IAV, with reports describing only altered fetal thymic transcription and reduced neutralizing antibody responses to IAV (*24*, *25*). Thus, the extent to which systemic maternal disease coordinates immune changes across the placenta and fetus, whether these responses scale with disease severity, and how they ultimately influence fetal development remain unclear.

The objective of our study was to determine the immunologic impact of a maternal infection with an IAV H1N1 2009 pandemic strain on the placenta and fetus in a pigtail macaque (*Macaca nemestrina*) model, with a focus on whether severity of maternal lung disease influenced these changes. While some of these molecular mechanisms have been identified in mice and rats, these animal models differ from human pregnancy in many respects, including a short gestation (3 to 5 weeks). Humans have a gestational period that is 8 to 10 times longer than these species, allowing for extended fetal brain development in utero. However, this long gestational period also increases placental and fetal exposure to seasonal waves of IAV infection. Nonhuman primates (NHPs) share many similarities with human pregnancy, including longer gestation, placental structure, and labor physiology (*29*, *30*). NHPs are also frequently used in IAV disease modeling due to their similarities in human clinical and pathological features (*31*, *32*). A few studies using pregnant NHPs have modeled IAV infection but were performed to specifically understand complement activation and infant brain development in African green monkeys and rhesus macaques, respectively (*33*, *34*). Thus, our central goal was to broadly characterize how IAV infection during pregnancy affects placental and fetal immunity in a pigtail macaque model, a highly translational model to human pregnancy.

## RESULTS

### Impact of maternal IAV disease on clinical outcomes and histopathology

Eleven pregnant pigtail macaques were inoculated with an IAV H1N1 2009 pandemic strain (A/CA/07/2009) in the third trimester (FLU cohort) using a combination of intranasal, intratracheal, intraocular, and intraoral routes with a total of 10^7.4^ plaque-forming units (PFU) (Fig. 1A). Results were compared to 16 historical control uninfected pregnant pigtail macaques (CTRL cohort; table S1), which received either subcutaneous medium inoculations (*N* = 9) (*35*) or saline inoculation into the choriodecidual space via catheters (*N* = 7) (*36*) for comparison against Zika virus or group B *Streptococcus* cohorts, respectively. For the FLU cohort, a Cesarean section and necropsies of the dam and fetus were performed 5 days after inoculation. For the CTRL cohort, the interval between inoculation and Cesarean section varied depending on the intended purpose of the design, ranging between 1 and 100 days (table S1). The mean gestational age on the day of Cesarean section was 133.7 days in the FLU cohort and 146.2 days in the CTRL cohort.

**Fig. 1.**
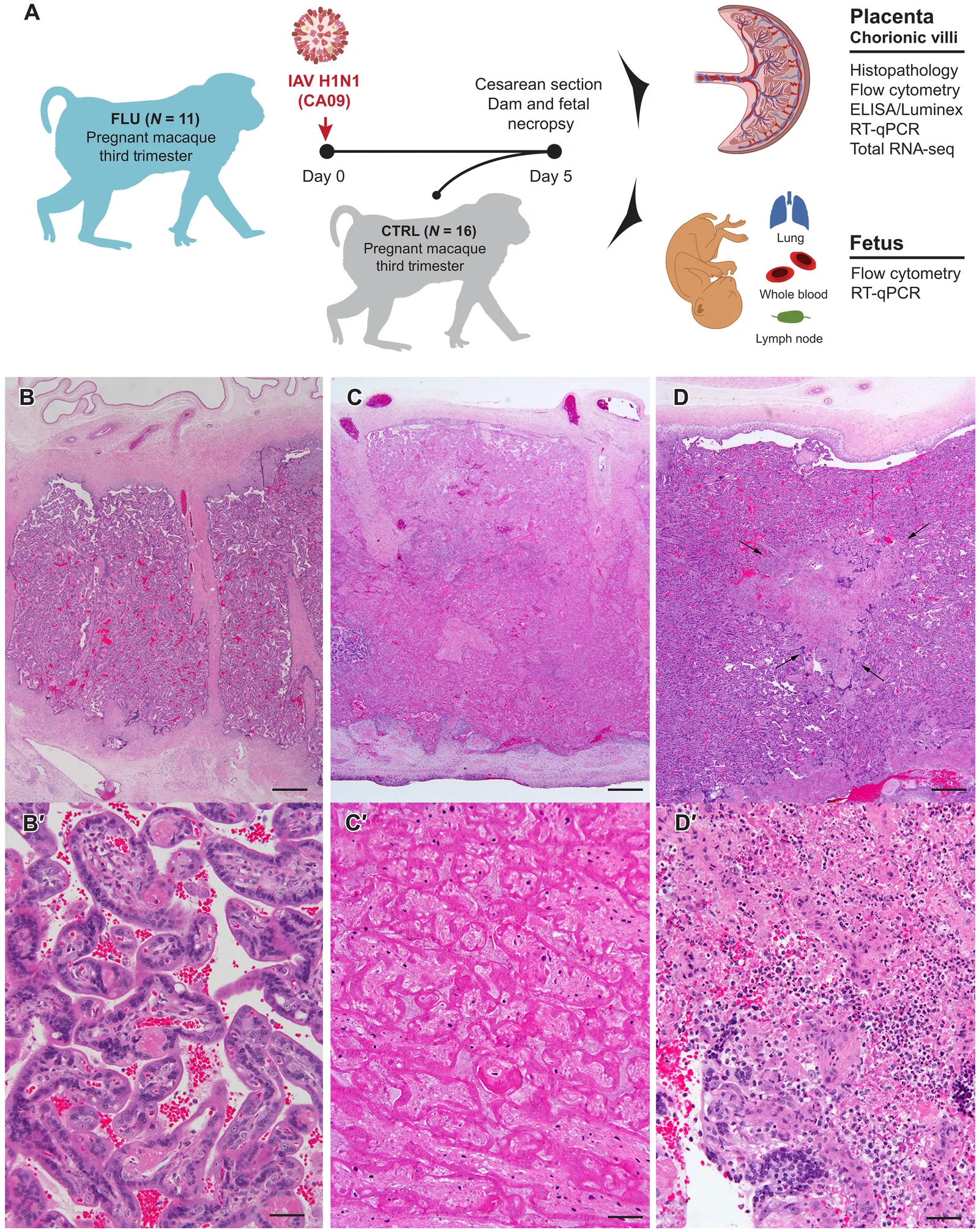
Experimental design and comparison of placental histopathology in the IAV and control groups. (**A**) Diagram of experimental design. “Experimental design” by S. Sun created in BioRender (https://BioRender.com/xnkqjsx) is licensed under CC BY 4.0. (**B** to **D**) Each pair of images shows a low-magnification image of the placental disc (fetal surface toward the top) in the top panel and a higher-magnification image of villous tissue in the bottom panel. [(B) and (B′)] Normal placental anatomy from a control animal showing no inflammation or necrosis. [(C) and (C′)] A focus of subacute infarction with coagulative necrosis and no active inflammation is shown from a representative animal in the FLU cohort. [(D) and (D′)] A focus of necrotizing villitis (highlighted by arrows) with neutrophilic inflammation and villous necrosis is shown from a representative animal in the FLU cohort. Scale bars, 500 μm [(B) to (D)] and 50 μm [(B′) to (D′)].

Clinical signs of maternal illness were documented using daily clinical scores and vital signs taken at the time of sedation: before inoculation, 1 and 3 days postinoculation (dpi), and at 5 dpi, the experimental end point (Table 1). Coughing, wheezing, increased respiratory effort, nasal discharge, and decreased appetite were common symptoms in 7 of 11 FLU animals (64%), while the remaining four did not show clinical symptoms. Animals that exhibited the most severe disease based on clinical scores were FLU8 and FLU9; of these animals, FLU9 experienced a stillbirth. Clinical scores were highest between day 3 and day 5 postinoculation. Uninfected CTRL animals did not show signs of respiratory disease. Histopathology of the maternal lungs and placentas was evaluated to determine if IAV inoculation induced influenza-associated pathology in either organ (Fig. 1, B to D, and table S2). Lung histopathology revealed pneumonia in all cases. Of the CTRL animals with histopathology data available, none had pulmonary inflammation.

**Table 1.** Clinical outcomes and maternal lung viral load. Clinical symptoms were scored on a three-point scale, with higher scores indicating worse disease and 0 indicating normal. The maximum clinical score was calculated by the sum of the highest recorded scores for each of the five clinical areas. D, day.

ID	Clinical symptoms	Stillbirth	Maximum clinical score	Maternal lung viral load (copies/mg)	Placenta viral load (copies/mg)
FLU1	Diarrhea: D4 and D5	No	3	2.43 × 10^6^	48.3
FLU2	Mild cough: D4	No	3	6.05 × 10^6^	0
	Dehydration: D5				
	Moderate wheezing: D5				
FLU3	None	No	0	8.58 × 10^5^	0
FLU4	None	No	0	2.22 × 10^3^	0
FLU5	Increased respiratory effort: D2	No	4	2.03 × 10^6^	0
	Mild cough: D3				
	Not eating: D3 and D4				
FLU6	None	No	0	5.69 × 10^5^	0
FLU7	Decreased appetite: D2	Yes	1	2.01 × 10^4^	0
FLU8	Sneezing: D2 and D3	No	6	1.41 × 10^6^	0
	Nasal discharge: D2 to D5				
	Increased respiratory effort: D2 to D4				
	Decreased appetite: D4				
	Diarrhea: D3 and D5				
FLU9	Nasal discharge: D2 and D4	Yes	4	3.17 × 10^3^	64.2
	Red nasal discharge: D3 and D5				
	Increased respiratory effort: D2 to D5				
	Decreased appetite: D4				
	Decreased responsiveness: D5				
FLU10	None	No	0	3.49	0
FLU11	Increased respiratory effort: D2 and D3	No	3	8.94 × 10^5^	0
	Nasal discharge: D3				
	Mild coughing: D3				

Stillbirths were diagnosed at the study end point in 2 of 11 FLU animals (18%; FLU7 and FLU9) but in no CTRL animals. The gestational ages of the stillborn fetuses were 134 days (FLU7) and 121 days (FLU9), corresponding to ∼74% of a full-term pigtail macaque pregnancy (∼172 days of gestation); the equivalent in a human pregnancy is estimated at 28 weeks of gestation based on an average of 38 weeks postconception until full term. In nearly 6 years of data from the Washington National Biomedical Research Center (WaNBRC) (1 January 2019 to 7 December 2025), stillbirth occurred in 2.6% (16 of 608) of pregnancies viable in a comparable time window (110 to 140 days). The rate of stillbirths in our FLU cohort was significantly greater than that observed in the breeding colony during this gestational age time window (*P* = 0.03).

Placental histopathology in the two stillbirth cases (FLU7 and FLU9) overlapped with that observed in other IAV-infected and some CTRL animals. None of the placentas demonstrated active inflammation of the chorioamniotic membranes. FLU animals had a greater but nonsignificant rate of placental infarction versus CTRL animals (FLU: 3 of 11, 27%; CTRL: 1 of 16, 6%; *P* = 0.08). Neutrophilic (necrotizing) villitis was observed frequently in both groups (FLU: 5 of 11, 45%; CTRL: 4 of 16, 25%; Fig. 1, C and D, and table S2), with no significant differences between FLU and CTRL cohorts. These findings are in line with other reports of high background rates of marginal placental infarctions and necrotizing villitis in macaque placentas (*37*–*39*).

### IAV H1N1 viral RNA detection in maternal lungs, placenta, and fetus

Reverse transcription quantitative polymerase chain reaction (RT-qPCR) was performed to measure IAV viral RNA (vRNA) in the maternal lungs, placenta, and fetal organs (Table 1). At 5 dpi, all FLU animals had high levels of IAV vRNA in their lungs, ranging from 2220 copies/mg to more than 6 million copies/mg; one exception was FLU10, which had an almost undetectable lung viral load (3 copies/mg). Maternal plasma was negative for IAV vRNA. In the placental chorionic villous tissue, IAV vRNA was detected in only two cases (2 of 11, 18%), both with a low copy number in the placenta (FLU1 and FLU9; Table 1). Of these, FLU9 additionally experienced stillbirth. Fetal tissue samples from the brain, brainstem, spinal cord, meninges, heart, lungs, liver, spleen, and thymus were negative for IAV H1N1 vRNA across the entire FLU cohort (table S3). Vertical transmission could not be confirmed in the FLU 9 stillbirth due to severe autolysis of the tissue, rendering the RNA quality insufficient for RT-qPCR. Overall, the maternal lung was characterized by a wide distribution of IAV viral load, infrequent detection of IAV vRNA in placental tissues, and no evidence for congenital infection.

### Placental and amniotic fluid cytokines

Cytokine responses in the placental disc and amniotic fluid were evaluated to determine if there was a correlation between maternal IAV infection status, viral load, or stillbirth. Chorionic villous tissue from the placenta disc of FLU animals had significantly greater concentrations of some inflammatory and antiviral cytokines than CTRL placentas, including interleukin-1β (IL-1β), IL-18, interferon-β (IFN-β), and granulocyte-macrophage colony-stimulating factor (GM-CSF) (all *P* ≤ 0.03; Fig. 2). The highest concentrations of IFN-α, IFN-β, IL-6, tumor necrosis factor (TNF), and GM-CSF within the FLU cohort were observed in both placentas with a low IAV viral load, of which one was a stillbirth case; however, the second stillbirth case had low cytokine concentrations. Several cytokines with low expression (IL-2, IL-10, IL-12p70, IL-13, IL-17A, and IL-23) were significantly higher in the CTRL versus FLU cohort (fig. S1). However, in many cases, these cytokine values fell below the working range of the Luminex assay. In the amniotic fluid many cytokine concentrations were significantly higher in the CTRL versus FLU cohort, including GM-CSF, IL-1β, IL-5, TNF, IFN-γ, IL-13, and IL-17A (all *P* < 0.05; fig. S2). Overall, these results indicate that perturbations in cytokines associated with maternal IAV infection were compartment specific, and elevated cytokines in the chorionic villous tissue were not always transported into the amniotic fluid compartment.

**Fig. 2.**
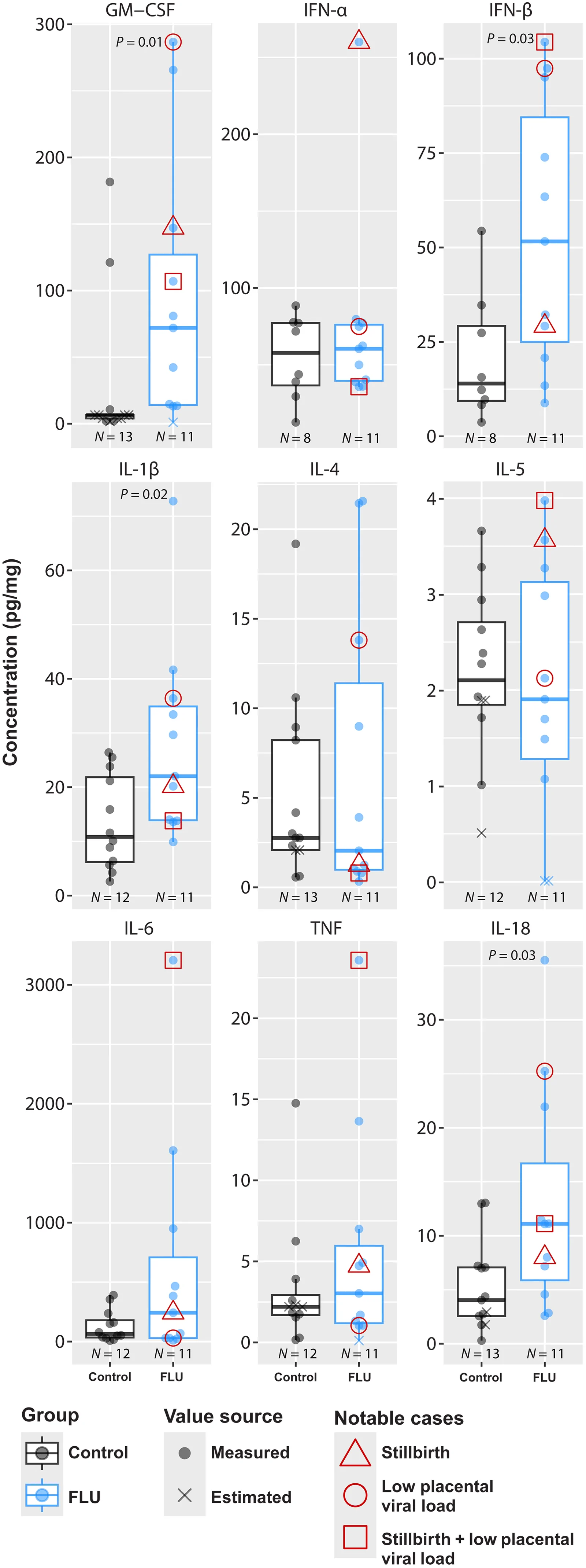
Placental cytokine concentrations in the IAV and control groups. Each panel shows the concentration of a cytokine, quantified by a multiplex Luminex assay, in chorionic villous tissue from the FLU (blue) and CTRL (black) cohorts. Values are either measured (dots) or estimated (×’s) by extrapolation of the standard curve. Estimated values were outside the range of the standard curve and were determined by the Luminex analysis software. The thick line indicates the median, and the bounding box indicates the first and third quartiles. A red triangle marks samples from animals that experienced a stillbirth. A red circle marks samples from animals that had a low placental viral load. A red square marks samples from animals that experienced a stillbirth and had low placental viral load. *P* values are shown using a Wilcoxon rank sum test comparing FLU versus CTRL cohorts. The *N* is shown at the bottom of each panel.

As several animals in the control group had lower gestational ages at inoculation and higher gestational ages at necropsy compared to FLU groups, we created age-defined subgroups of animals and reran our analyses (table S4 and fig. S3). These gestational age–matched CTRL subgroup for the FLU inoculation and delivery gestational age groups had approximately the same average gestational age at inoculation/necropsy and were restricted within a comparable developmental period. When analyses were restricted to gestational age–matched CTRL animals, nearly all cytokines remained significant (*P* < 0.05), except for several within the inoculation age subset of amniotic fluid (i.e., IFN-β, IL-1β, and TNF; fig. S3).

### Placental immune cell frequencies

To determine whether maternal IAV H1N1 infection was associated with changes in the innate and adaptive immune cell composition of the placental disc, we performed flow cytometry–based immunophenotyping. The frequency of classical monocytes among live leukocytes was significantly decreased in the placental disc of the FLU versus CTRL cohort (*P* = 0.002; Fig. 3). There was no relationship between stillbirth and the frequency of immune cell populations in the placenta. When using the gestational age–matched CTRL cohorts, the lower classical monocyte frequencies in FLU versus CTRL remained significant (*P* < 0.05; fig. S4). These data indicate that a maternal IAV infection is associated with a reduction in classical monocyte frequency.

**Fig. 3.**
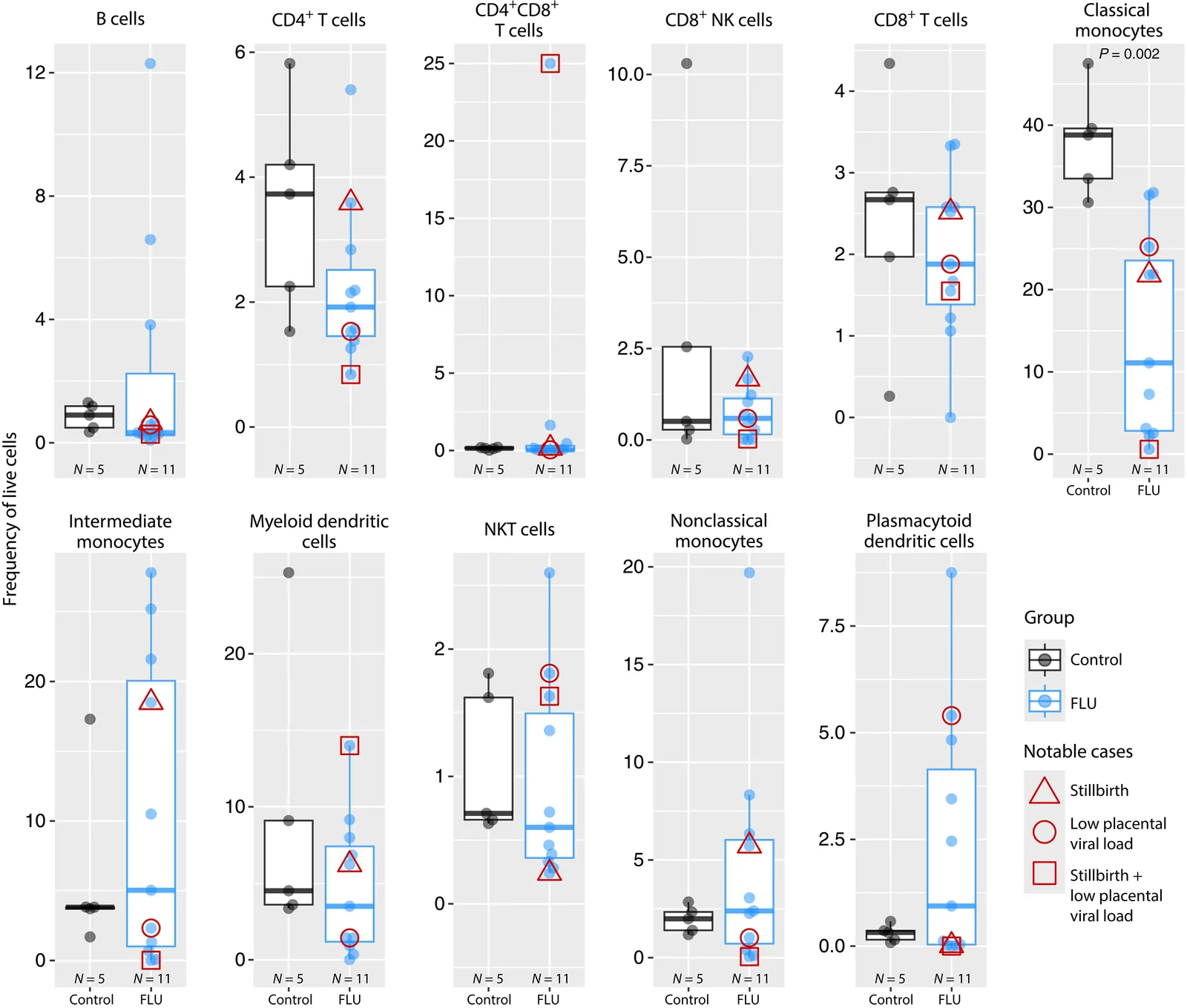
Placental immunophenotyping in the IAV and control groups. Each panel shows the frequency of the defined cell population as a percentage of live cells, in chorionic villous tissue of the FLU (blue) and CTRL (black) cohorts. The thick line indicates the median, and the bounding box indicates the 1st and 3rd quartiles. A red triangle marks samples from animals that experienced stillbirth. A red circle marks samples from animals that had a low placental viral load. A red square marks samples from animals that experienced a stillbirth and had low placental viral load. *P* values are shown using a Wilcoxon rank sum test comparing FLU versus CTRL cohorts. Significance is considered for *P* < 0.05 but is shown if 0.05 ≤ *P* ≤ 0.1 due to the modest sample size. The *N* is shown at the bottom of each panel.

### Placental transcriptional profile

To determine whether a maternal IAV infection altered the transcriptional signature in the placental disc, we performed total RNA sequencing (RNA-seq) on chorionic villous tissues and validated our results by direct gene counting using a NanoString nCounter assay. Note that all animals for which we had transcriptional data are contained within the gestational age–matched CTRL subgroup (table S4). A multidimensional scaling plot was created to visualize the normalized RNA-seq gene counts and demonstrated a clear division between the two cohorts [CTRL (blue dots) and FLU (red symbols); fig. S5]. In the placental chorionic villous tissue, 185 genes were up-regulated, and 178 genes were down-regulated relative to the gene expression of the CTRL group (≥ or ≤1.5-fold, *P* < 0.05; Fig. 4A). Inspection of the up-regulated genes revealed a notable up-regulation of IFN-stimulated genes (ISGs) including *IFIT3*, *MX1*, and *OAS2* in placentas from FLU animals relative to CTRL placentas. A gene set enrichment analysis revealed multiple significant, positively enriched pathways related to the type I IFN response and the negative regulation of viral processes such as genome replication and viral entry in FLU versus CTRL animals (Fig. 4B). Digital gene counting using a 770-plex NanoString nCounter panel supported these results with a similar notable up-regulation of ISGs (fig. S6) and strong correlation with total RNA-seq gene counts in both the FLU and CTRL groups (*r* = 0.8, *P* < 2.2 × 10^−16^ for both; fig. S7).

**Fig. 4.**
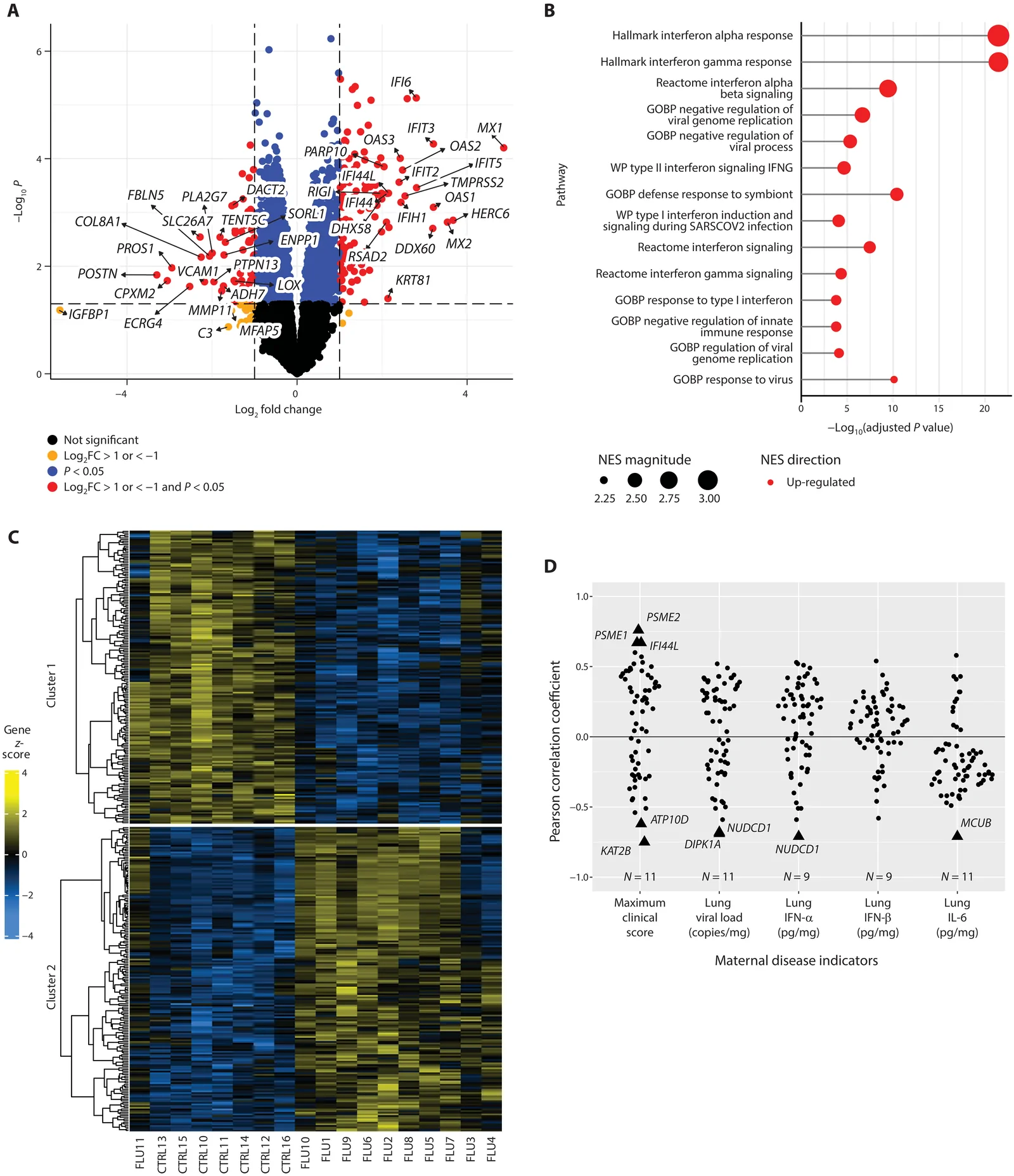
Placenta total RNA-seq analysis in the IAV and control groups. (**A**) The volcano plot shows log_2_ fold change versus –log_10_(*P* value) for FLU (*N* = 11) versus CTRL (*N* = 7). Red dots mark significant genes with |log_2_ fold change| (|log_2_FC|) ≥ 1; blue dots mark genes with |log_2_FC| < 1 but *P* < 0.05; gold dots indicate nonsignificant genes with |log_2_FC| ≥ 1; black dots indicate nonsignificant genes with |log_2_FC| <1. (**B**) Gene set enrichment analysis of significant GO terms (*P* < 0.05) displays the normalized enrichment score (NES) for each category; false discovery rate (FDR) values reflect correction for multiple testing (**C**) Heatmap of normalized gene counts from placental total RNA-seq comparing FLU-exposed (*N* = 11) and CTRL (*N* = 7) cohorts. Each column represents an individual placental sample, and each row represents a single gene. Yellow-to-blue color intensity reflects the gene-level *z*-score, calculated using the mean expression value across all animals for each gene. Unsupervised hierarchical clustering was performed on the top 500 differentially expressed genes identified in FLU placentas, using Euclidean distance and Ward’s linkage method. (**D**) Pearson correlation analysis between a select panel of 64 placental genes (listed in figs. S8 and S9) and key metrics of maternal IAV disease severity: maximum clinical score, lung viral load, lung IFN-α concentration, lung IFN-β concentration, and lung IL-6 concentration. Cytokine concentrations and viral load were log_10_-transformed before analysis. The Pearson correlation coefficient (*R*) is plotted for each gene-metric pair. Each dot/triangle represents a correlation that was run between a maternal disease indicator (specified by the column) and the normalized gene count of a given gene using FLU animals. The number of FLU animals used to run each correlation is consistent for each column, and the *N* is shown at the bottom of the column. A total of 320 distinct correlations were run. Significant correlations (unadjusted *P* < 0.05) are shown as triangles, and nonsignificant correlations (unadjusted *P* ≥ 0.05) are shown as dots. Genes with statistically significant correlations are labeled.

Next, we compared bulk transcriptomic changes in the placenta between the FLU and CTRL cohorts to identify pathways differentially expressed by IAV. Hierarchical clustering yielded two distinct clusters of differentially expressed genes: cluster 1 (down-regulated genes, FLU versus CTRL; Fig. 4C) and cluster 2 (up-regulated genes, FLU versus CTRL; Fig. 4C). Overrepresentation analysis (ORA) was performed to identify Gene Ontology (GO) terms enriched in both clusters. No significant GO terms were identified in cluster 1. However, inspection of genes in cluster 1 revealed down-regulation in the FLU group of multiple genes involved in ubiquitination, proteasome function, unfolded protein response, metabolism, and cell cycle/DNA repair (fig. S8). To determine whether this profile might reflect the induction of a viral-associated integrated stress response (ISR), we analyzed the expression of two key pathway genes: *EIF2AK2* (ISR activator) and *ATF4* (ISR effector). Of these genes, *EIF2AK2* expression was significantly up-regulated in the placenta (log_2_ fold change = 1.24, *P* < 0.05; fig. S8). In cluster 2, significantly enriched GO terms in the FLU cohort were related to type I and II IFN signaling, viral genome regulation, and antiviral defense processes that featured ISGs (figs. S9 and S10). A notable outlier was FLU11, which had a transcriptional profile more similar to that of CTRL animals. Placental transcriptomics of FLU3 and FLU4 were intermediate between the FLU and CTRL cohorts. Collectively, transcriptional analysis of the placental chorionic villous tissue from FLU animals revealed a notable up-regulation of antiviral innate immune genes and down-regulation of critical cellular functions (cell cycle, metabolism, and ubiquitination) consistent with an antiviral and ISR response, regardless of the severity of disease in the maternal lung.

### Placental correlations with maternal lung disease indicators

We hypothesized that placental gene expression might correlate with clinical score, lung viral load, or an immunologic correlate of IAV-associated lung disease, such as an inflammatory cytokine (IL-6) or the antiviral type I IFN response (lung IFN-α and IFN-β). A panel of 64 key genes derived from the hierarchical clustering model was selected as key genes for the correlation analysis (listed in figs. S8 and S9). Few correlations between these outcome metrics emerged (Fig. 4D). Evaluating correlations between maternal disease indicators and placental cytokines or immune cell frequencies similarly yielded almost no significant correlations (figs. S11 and S12). Overall, these data suggested a weak correlation between IAV-associated maternal lung disease indicators and placental gene expression or cytokine concentrations.

### Fetal immune cell frequencies in lung, lymph node, and blood

Next, we examined fetal lung, lymph nodes, and blood compartments using flow cytometry–based immunophenotyping. In these fetal organs, there was a coordinated perturbation of innate immune cells and T cells in the FLU versus CTRL cohorts. In the fetal lung, the frequencies of intermediate monocytes, nonclassical monocytes, plasmacytoid dendritic cells, and CD4^+^ T cells were significantly higher in the FLU cohort compared to the CTRL cohort (*P* ≤ 0.04; Fig. 5). A similar pattern was evident in the fetal lymph nodes, with a significantly higher frequency of intermediate monocytes (*P* = 0.02). Additional immune populations in the fetal lymph node had a higher but nonsignificant distribution of natural killer T cells (NKT cells), nonclassical monocytes, and plasmacytoid dendritic cells in FLU versus CTRL animals (all *P* = 0.06; Fig. 5). In fetal whole blood, there was a higher frequency of NKT cells in the FLU cohort, although this result was not significant (*P* = 0.07; Fig. 5). When analyses were restricted to CTRL animals so that the mean delivery gestational age was within ±1 week of FLU animals, the higher frequencies of nonclassical and intermediate monocytes in the fetal lungs remained significant (*P* < 0.05; fig. S13). Overall, these differences suggest that maternal IAV H1N1 infection perturbs populations of fetal innate and adaptive immune cells in organs, lymphoid compartments, and blood, even in the absence of vertical transmission.

**Fig. 5.**
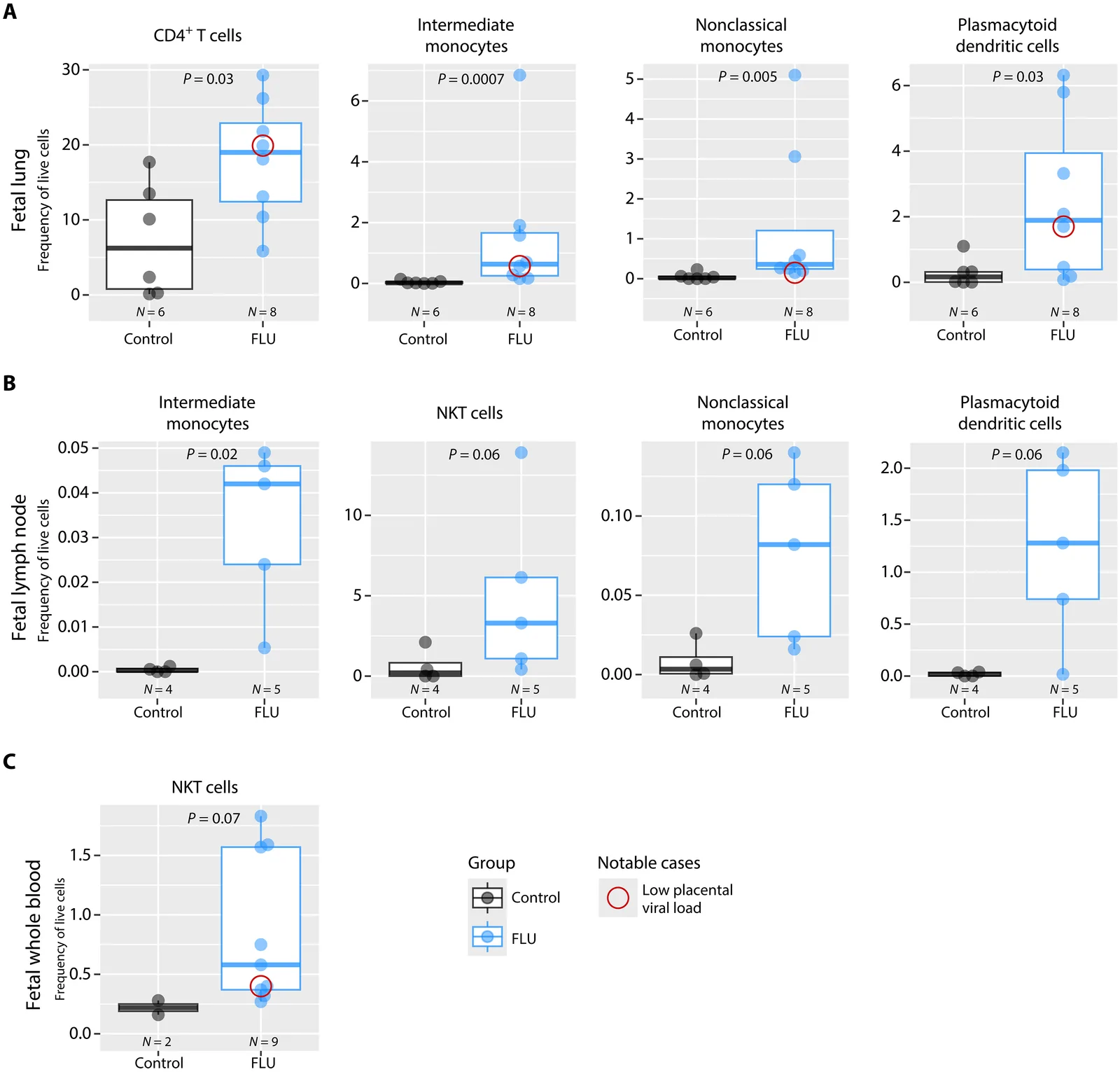
Fetal immunophenotyping in the IAV and control groups. Each panel shows the frequency of the defined cell population as a percentage of live cells, in fetal lung (**A**, top row), fetal lymph node (**B**, middle row), and fetal whole blood (**C**, bottom row) of the FLU (blue) and CTRL (black) cohorts. The thick line indicates the median, and the bounding box indicates the first and third quartiles. A red circle marks samples from animals that had a low placental viral load. Fewer number of observations in a group reflects inability to obtain sufficient samples or cells to perform the assay. Immunophenotyping was not done on fetal samples from stillbirth cases. The *P* values were generated using a Wilcoxon rank sum test and are indicated at the top of each panel. Significance is considered for *P* < 0.05 but is shown if 0.05 ≤ *P* ≤ 0.1 due to the modest sample size. The *N* is shown at the bottom of each panel. Additional results are in figs. S14 to S16.

### Fetal correlations with maternal lung disease indicators

Next, we performed an exploratory analysis to examine correlations between maternal lung disease indicators with fetal immune cell populations, motivated by a growing literature linking maternal respiratory infections with dysregulated fetal immunity (fig. S17) (*40*–*44*). These analyses were not adjusted for multiple comparisons given the modest sample size and number of correlations performed. In contrast to the few correlations identified in the placenta, there were multiple significant correlations between IAV-associated maternal lung disease and fetal immune cell populations that remained consistent across diverse organ and fluid compartments (lungs, lymph nodes, and whole blood; fig. S17). A common pattern emerged of significant, positive correlations between metrics of maternal disease severity and higher frequencies of fetal T cell populations (CD4^+^, CD8^+^, CD4^+^CD8^+^ T cells) or plasmacytoid dendritic cells, which are major producers of type I IFNs (all *P* < 0.05). Furthermore, maternal lung type I IFN concentrations were significantly and positively correlated with CD8^+^ T cell frequencies in the fetal blood and lungs. Maternal disease indicators were negatively correlated in some cases with fetal monocyte populations (classical, nonclassical, and intermediate) and myeloid dendritic cells. Collectively, these data suggest that maternal IAV disease is associated with perturbations in immune cells across multiple fetal tissue and fluid compartments.

## DISCUSSION

The placenta is a critical immunological gatekeeper during pregnancy, balancing its essential roles in nutrient and oxygen exchange with the need to protect the fetus from invading pathogens. Our data suggest that placental IAV exposure induces a cascade of immune defenses, including a robust placental type I IFN response (IFN-β). These responses are characteristic of a rapid antiviral defense state, in which the placenta prioritizes pathogen restriction over immune quiescence to protect and nourish the fetus. This placental antiviral response occurred independently of maternal lung disease severity, supporting the concept that the placenta transitions into a defensive immune state in response to maternal infection, effectively shielding the fetus from infection at the potential cost of immune injury to the placenta and fetus, and stillbirth.

To our knowledge, this study provides the first demonstration that maternal IAV infection can broadly perturb the fetal immune landscape in a manner linked to maternal metrics of immunity and disease in an NHP model. As vertical transmission was not detected in our experiments, the data suggest that immunologic, rather than virologic, signals are responsible for the profound changes in the fetal immune landscape observed after in utero exposure to maternal IAV infection. Notably, fetuses in the FLU cohort exhibited significantly higher frequencies of multiple T cell populations and reduced frequencies of monocytes compared with CTRLs, with these changes evident across multiple organs and fluid compartments and correlated with key metrics of maternal IAV disease. Although the mechanisms by which host-pathogen interactions shape fetal immune development and long-term health remain incompletely understood, maternal disease severity represents a potentially modifiable determinant of these outcomes. We also note that several articles in rodent models have previously shown a link between maternal IAV infection and perturbations in fetal thymic transcriptome (*24*), hematopoietic development (*45*), and lymphoid organ compartmentalization (*46*).

Altered fetal immunity has previously been described following in utero exposure to maternal HIV infection, even in the absence of maternal–fetal viral transmission. Perinatally HIV-exposed but uninfected infants have been shown to have a lower frequency of naïve CD4^+^ and CD8^+^ T cells, among other abnormalities in immune activation, compared to perinatally HIV-unexposed infants (*47*–*50*). Similar findings of a perturbed fetal immune landscape have been reported in cases of maternal severe acute respiratory syndrome coronavirus 2 (SARS-CoV-2) infection. Perturbed monocyte cell populations with increased NK cell and regulatory T cell frequencies were reported in uninfected neonates exposed to maternal SARS-CoV-2 (*41*, *44*). These changes in fetal immunity may represent an intermediate biological state that accompanies pathways leading to abnormal neurodevelopment, as described in many cohorts of adults exposed to maternal influenza and other infections during fetal life (*15*, *16*, *21*, *22*, *51*, *52*).

Stillbirth occurred in two animals in the FLU cohort, an outcome that was unexpected based on the CTRL cohort and historical colony experience. The placental antiviral program triggered by IAV H1N1 is known to promote inflammation and tissue injury during acute viral infections and has been directly implicated in the pathogenesis of stillbirth in mouse models of congenital Zika virus infection (*53*–*56*). Whether a viral-associated ISR may have also contributed to stillbirth through translational repression and maladaptive stress signaling is unknown. We are cautious to causally attribute these stillbirths solely to the placental type I IFN or ISR response for several reasons. A spectrum of placental pathology was observed in FLU animals, with similar lesions (i.e., infarction and necrotizing villitis) also present in several CTRL placentas from clinically healthy animals. Background placental pathology in macaques is known to be relatively high, with prior studies documenting marginal infarctions and villitis as common findings even in uncomplicated pregnancies (*37*–*39*). Moreover, unlike human stillbirth evaluations, which routinely involve extensive histopathologic sampling across the entire placental disc, the placental tissue in this study was allocated to multiple downstream immunologic and transcriptomic analyses, thereby limiting a comprehensive histopathologic examination. Together, these factors limit our ability to assign a specific pathological mechanism to the two stillbirths, underscoring the need for cautious interpretation while highlighting a biologically plausible link between antiviral placental immune activation and adverse perinatal outcomes.

The primary strength of our study lies in the translational nature of the NHP model, which closely recapitulates human pregnancy, enabling modeling of an IAV infection in the context of pregnancy. NHPs are highly similar to humans in their genetics, anatomy, physiology, immune responses, and pregnancies (*29*). Macaques have a long gestational period and a placental architecture similar to that of humans. Another strength of the pregnant NHP model of IAV infection is the ability to study antiviral immune responses at a defined time after infection onset, which is not possible during human pregnancy. Further, this study was deliberately conducted in the third trimester of pregnancy, a time when pregnant women experience the greatest maternal morbidity and mortality (*57*, *58*). NHP studies excel at identifying integrated physiologic outcomes that cannot be replicated in other models, providing an essential translational context for human pregnancy. Defining molecular mechanisms in NHP models is constrained by biology and ethics, in contrast to in vitro or mice models, which offer considerably greater experimental manipulation. These features of the pregnant NHP model of IAV infection demonstrate the translational relevance of this model and the marked impact of our findings, while also providing possible mechanistic insights for future studies.

This study has several limitations, primarily related to ethical constraints involved with NHP studies and the modest sample size. We did not study end points earlier or later than 5 days postinfection, preventing assessment of the trajectory of the antiviral response in the maternal lungs and placenta. We used multiple routes of viral inoculation to ensure productive disease; experiments using lower inocula delivered via aerosol would more closely recapitulate IAV transmission in humans. Examining multiple end points was not feasible with our limited sample size, but future studies could investigate alterations in fetal immune regulation after maternal IAV disease resolution and the potential for development of an ISR in the placenta. While the stillbirths are notable cases, the study’s conclusions are drawn from patterns across the entire infected cohort. Our CTRL cohort consisted of historical uninfected controls used in prior studies. While housing conditions, sample collection, and experimental protocol remained consistent across all animals, the use of historical controls provides challenges in accurately controlling confounding variables, such as the gestational age at the inoculation and necropsy time points. After performing analyses using a subset of the CTRL cohort to account for these variations in gestational age, we found that most of our conclusions remained significant, even with reduced statistical power.

Together, these findings position the placenta as an active and autonomous organ that mounts a robust type I IFN defense against IAV, which may partly explain the rarity of vertical transmission. In contrast, fetal immunity appears to be sensitive to maternal disease severity, with perturbations in fetal T cells and monocytes across multiple fetal compartments occurring in a coordinated response. An advantage of the NHP model is the ability to interrogate immune changes in multiple fetal tissues to a maternal respiratory virus infection, similar to studies in human SARS-CoV-2 studies (*40*, *43*). Despite public health recommendations for pregnant women to receive the seasonal IAV vaccine (*59*), only half of pregnant women in the US typically receive it each year (*60*). Seasonal IAV vaccination is known to reduce maternal morbidity, mortality, and stillbirth (*11*, *61*–*65*). Whether mitigation of maternal IAV disease through vaccination preserves the normal trajectory of fetal immune development is an important question.

## MATERIALS AND METHODS

### Study design

Two groups of female pigtail macaques (*M. nemestrina*) were inoculated using a combination of intranasal, intratracheal, intraocular, and intraoral routes with 10^7.4^ PFU of IAV [A/California/07/2009 (H1N1)], reflecting a dosing method previously used in a study of cynomolgus macaques (*Macaca fascicularis*), which resulted in a productive infection (*66*). Experimental groups consisted of IAV H1N1–infected pregnant (*N* = 11) and uninfected pregnant controls (*N* = 16; table S1). IAV inoculation occurred in the early third trimester to model the time in pregnancy when IAV H1N1 infections are thought to produce the greatest maternal lung disease and pregnancy complications (*13*, *14*). Uninfected controls in this study consisted of pregnant adult pigtail macaques used in prior published studies from two cohorts: (i) control studies catheterizing the amniotic cavity and inoculating saline into the choriodecidual space and/or amniotic fluid; (ii) control studies inoculating medium subcutaneously to mimic procedures in a Zika virus experimental cohort (*35*, *36*). Ethical constraints limit the availability of large, dedicated control cohorts for each experimental study. Thus, historical controls were used with consistent husbandry conditions, necropsy and processing protocols, and experimental protocols to minimize potential confounding variables. Animals in the WaNPRC were not exposed to seasonal IAV infections and thus do not have preexisting immunity to IAV.

### Ethics statement and animal care

All animal experiments used in this study were approved by the University of Washington Institutional Animal Care and Use Committee (protocol# 4165-03, last approved 22 July 2025) and in compliance with the US Department of Health and Human Services Guide for the Care and Use of Laboratory Animals and Animal Welfare Act. All animal experiments were performed at the WaNPRC.

Macaques inoculated with IAV were observed daily for clinical symptoms of influenza disease, which were scored on a three-point scale to reflect responsiveness of the animal, nasal or ocular discharge, cough, respiratory effort, food consumption, and fecal consistency; higher scores reflected disease, with a 0 in each category indicating a normal assessment. We chose a predefined study end point of 5 dpi to capture immune responses near the expected peak of disease (*67*, *68*). A Cesarean section was performed at the end point, and placentas were extracted to sample chorionic villous tissue. The dam was euthanized using a barbiturate overdose while under anesthesia. The fetus was euthanized using a barbiturate overdose, followed by exsanguination. These methods are approved by the 2007 American Veterinary Medical Association Guidelines on Euthanasia. Necropsies were performed on both the dam and the fetus to obtain samples for transcriptomics, immune assays, and histopathology. Random sections of the placental membrane, chorionic disc, and umbilical cord were evaluated for pathology. In one stillbirth case, FLU9, the fetus was 1 to 2 days postmortem at the time of Cesarean section. Pathologists reported that severe autolysis of the tissue had occurred. Samples were not collected from the fetus because the tissue quality would likely be too low to perform meaningful assays.

### IAV H1N1 virus cultivation and quantification

We used an IAV strain [A/California/07/2009 H1N1 influenza virus (CA09)] that was obtained from the World Reference Center for Emerging Viruses and Arboviruses at the University of Texas Medical Branch at Galveston. Before culturing the virus, Madin-Darby canine kidney (MDCK) cells (American Type Culture Collection, Manassas, VA, USA) were cultured in Dulbecco’s modified Eagle’s medium supplemented with 10% fetal bovine serum, 25 mM Hepes, penicillin (100 U/ml), and streptomycin (100 μg/ml). Cells were incubated at 37°C and 5% CO_2_ and confirmed to be free of mycoplasma with the MycoAlert mycoplasma detection kit (LT07-318, Lonza) before use for virus cultivation. Subconfluent monolayers of MDCK cells in T150 flasks were washed with phosphate-buffered saline (PBS) and infected at a multiplicity of infection of 0.5 (5 ml of serum-free minimum essential medium) at 37°C and 5% CO_2_. After 1 hour, the medium was removed, cells were washed with 1× PBS, and the medium was replaced. The supernatant was harvested after 36 hours and centrifuged at room temperature to remove cellular debris (1800 rpm for 5 min, followed by 3000 rpm for 10 min). Virus stock was frozen at −80°C. The viral titer of each stock was quantified using a median tissue culture infectious dose (TCID_50_) assay with hemagglutination readout and converted to PFU per milliliter (*69*). To transform TCID_50_ per milliliter into PFU per milliliter, TCID_50_ per milliliter was multiplied by 0.7. Reagents were obtained from Thermo Fisher Scientific unless otherwise stated (Waltham, MA).

### RNA isolation and RT-qPCR

Tissue samples were kept in RNAlater Stabilization Solution (#15596018, Thermo Fisher Scientific) before homogenization in Precellys homogenizing tubes (#1011152, Cayman Chemical, Ann Arbor, MI, USA) with the Precellys Evolution machine (Bertin Technologies, Montigny-le-Bretonneux, France). RNA was extracted from homogenates using the RNeasy Mini Kit (#74106, QIAGEN) and treated with deoxyribonuclease (#79256, QIAGEN). RNA was quantified using the NanoDrop 2000 spectrophotometer (#ND-2000, Thermo Fisher Scientific) and the Qubit 4 fluorometer (#Q33238, Invitrogen). RNA quality was evaluated by measuring the RNA integrity number (RIN) with the 4200 TapeStation System (#G2991BA, Agilent).

To quantify IAV vRNA levels from different sample origins, we used RT-qPCR and a WHO-approved primer/probe set (*70*). cDNA was generated from RNA isolates using an iScript Select cDNA synthesis kit (#1708897, Bio-Rad) using H1N1 HA-specific forward primer following the manufacturer’s instructions. qPCR was done with H1N1 HA-specific primers and probes (forward, GGGTAGCCCCATTGCAT; reverse, AGAGTGATTCACACTCTGGATTTC; probe: 5′-[FAM] TGGGTAAATGTAACATTGCTGGCTGG [TAMRA]-3′) using the TaqMan Fast Advanced Master Mix (#444964, Thermo Fisher Scientific) and a QuantStudio 3 machine (#A28132, Thermo Fisher Scientific) following the manufacturer’s instructions. Samples were run in triplicate.

An external plasmid standard curve was used to determine vRNA quantities with the lower limit of quantification being three copies per reaction. A negative control with no template added was used to assess the possibility of contamination. Amplification was defined as fluorescent activity exceeding a threshold of 0.1. Negative results were defined as any sample that did not amplify or had a *C*_t_ value greater than 38 cycles. Positive results were defined as any amplified sample with a *C*_t_ value less than 38 cycles for all three replicates. Samples with low positive results (*C*_t_ > 35) had RNA reextracted from a new sample aliquot, and RT-qPCR was run two more times in triplicate to verify results, totaling nine replicates. Copies of vRNA per milligram of tissue were calculated on the basis of tissue weight, RNA concentration, RNA volume, and volume of cDNA sample input.

### Cytokine quantification

ProcartaPlex NHP multiplex kits were used to quantify cytokines from lysates or fluids (Thermo Fisher Scientific). Tissue lysis and homogenization were performed using an Invitrogen ProcartaPlex Cell Lysis Buffer (#EPX-99999-000, Thermo Fisher Scientific) with the addition of a protease inhibitor cocktail (#PI78430, Thermo Fisher Scientific) using the Precellys Evolution system, as previously described. Type I IFNs (IFN-α and IFN-β) were quantified using the cynomolgus IFN-β and cynomolgus/rhesus IFN-α enzyme-linked immunosorbent assay (ELISA) kits (#46415-1 and #46100-1, respectively, PBL Biosciences, Menlo Park, CA, USA). All protocols followed the manufacturer’s guidelines. The Luminex analysis software estimated cytokine concentrations that fell outside the range of the standard curve.

### Immunophenotyping

Immunophenotyping by flow cytometry was performed to determine the frequencies of immune cells in the placenta and fetal lung, mesenteric fetal lymph node, and fetal blood. Tissues from the placental disc and fetal organs were dissociated into single-cell suspensions and assessed for viability using acridine orange/propidium iodide (Nexcelom Bioscience LLC). Then, samples were stained with a panel of antibodies in Brilliant Stain Buffer (BD Biosciences) for 20 min to identify immune cells as previously described (*71*). Antibodies used included BD Biosciences: CD3 (SP34-2)–BV650, CD8 (RPA-T8)–BUV395, CD11b (ICRF44)–APC–Cy7, CD11c (SHCL-3)–phycoerythrin (PE), CD45 (D058-1283)–PE–CF594, CD86 (IT2.2)–PE–Cy5, and γδ T cell receptor (B1)–fluorescein isothiocyanate; BioLegend: CD4 (OKT4)–BV605, CD14 (M5E2)–BV785, CD16 (3G8)–Alexa Flour 700, CD20 (2H7)–BV570, and human leukocyte antigen–DR (L243)–BV711; Invitrogen: CD123 (6H6)–eFlour 450 and LIVE/DEAD Fixable Aqua for 405-nm excitation; Beckman Coulter: CD159a (Z199)–PC7; and Miltenyi: CD66 (TET2)–APC. The staining protocol proceeded in three steps: LIVE/DEAD Fixable Aqua, then all antibodies other than CD11c and CD123, and lastly CD11c and CD123. After staining, samples were fixed with 1% paraformaldehyde and analyzed on a Symphony A3 flow cytometer (BD Biosciences) using FACS Diva 8 software (BD Biosciences). Samples were analyzed using FlowJo 10.9.0 (FlowJo LLC). All events were initially gated on forward scatter (FSC) singlets, CD45^+^ leukocytes, live cells, and then on FSC-A and side scatter–A profiles. Populations were defined using canonical markers as described in table S5. Representative gating strategies are shown for granulocytes (fig. S18), lymphoid cells (fig. S19), and monocytes (fig. S20). Populations were excluded from analysis if they did not meet the minimum threshold of >100 cells in their parent gate.

### Total RNA-seq library preparation and analysis

Total RNA-seq was performed on chorionic villous tissue from the placenta. We used 100 ng of RNA with RIN > 3 as input for RNA library prep using the KAPA RNA HyperPrep kit with RiboErase HMR (#08098131702, Roche Diagnostics Corporation, Indianapolis, IN, USA). The kit was not only designed for human, mouse, and rat samples but also effectively reduced ribosomal RNA in our NHP RNA isolates. Libraries were prepared following the manufacturer’s standard protocol. Library quantity and quality were evaluated using Qubit Fluorometer (Thermo Fisher Scientific) and TapeStation (Agilent Technologies, Santa Clara, CA, USA). Constructed libraries were sequenced on the NovaSeq 6000 Sequencing System (#20012850, Illumina, San Diego, CA, USA), which produced 2 × 100 nucleotide-stranded paired-end reads.

Raw sequences were aligned to the *M. nemestrina* reference genome from Ensembl (Mnem_1.0, INSDC Assembly GCA_000956065.1) using STAR in Partek Flow, a bioinformatics software suite (Partek, Chesterfield, MO, USA). Ensembl gene symbol conversions were used to ensure consistency across all gene IDs. As the quality score was greater than 35 across all samples, no bases were trimmed, and 100% of sequencing bases overlapped with read lengths of the reference genome. The raw count matrix was downloaded from Partek. Subsequent statistical processing and analysis of count data were performed within the R statistical computing environment (R version 4.4.0). Gene counts were filtered by a row mean of 10 or greater, then normalized using edgeR to implement TMM normalization. Counts were transformed into log_2_ counts using voom, a function in the limma package that modifies RNA-seq data to create a normalized count matrix (*72*, *73*). Differential expression (DE) analysis compared placental samples from IAV-infected animals with those from uninfected controls. Comparisons were based on a linear model fit for each gene using limma. The criteria for all DE analyses were an absolute fold change of 2 and a *P* < 0.05. Adjusted *P* values were calculated using the Benjamini-Hochberg method to reduce the false discovery rate (FDR). Gene set enrichment analysis was conducted across the comparison group using fgsea with the Broad Institute Molecular Signatures database (*74*).

Hierarchical clustering analysis, an unsupervised machine learning approach, was performed to group total RNA-seq profiles based on their similarity in Euclidean distance using the hclust function in R. Ward’s linkage was used to minimize variance within each cluster. The optimal number of clusters was determined using the elbow method. The resulting hierarchy was visualized using a dendrogram. Last, an ORA was performed to determine if predefined sets of genes in GO pathways were enriched in the cluster of differentially expressed genes obtained from the hierarchical clustering analysis using the clusterProfiler package in R.

### NanoString nCounter

To validate our results from bulk RNA-seq, we chose a quantitative multiplex method of direct digital counting of gene transcripts using the NanoString nCounter Analysis Systems (NanoString Technologies Inc., Seattle, WA, USA). RNA extracted from the chorionic villous tissues of the placenta was analyzed using the NanoString nCounter NHP Immunology V2 gene expression code set (#11500276, NanoString Technologies Inc.). RNA hybridization reactions were performed according to the manufacturer’s protocol. Probe-target RNA complexes from all reactions were processed and immobilized on nCounter cartridges using the nCounter Prep Station. Gene transcripts were quantified with the nCounter Digital Analyzer (#MAN-C0035-07, NanoString Technologies Inc).

### Statistical analyses

All statistical analyses were performed in RStudio (using R version 4.4.0) or nSolver (nCounter data only), both of which use the R programming language. A *P* value of less than 0.05 was considered significant, and that of 0.05 to 0.1 was reported considering the modest sample size. Groups were compared using nonparametric tests (Wilcoxon rank sum) and Fisher’s exact tests. Pearson’s correlation coefficient was used to calculate correlations between two variables using unadjusted *P* values. Data visualizations were done using ggplot2, ggpmisc, and ComplexHeatmaps packages and their derivatives (*75*, *76*).

## References

[1] T. Van Le, L. T. Phan, K. H. K. Ly, L. T. Nguyen, H. T. Nguyen, N. T. T. Ho, T. X. Trinh, N. N. Tran Minh, Fatal avian influenza A(H5N1) infection in a 36-week pregnant woman survived by her newborn in Sóc Trăng Province, Vietnam, 2012. Influenza Other Respi. Viruses 13, 292–297 (2019). 10.1111/irv.12614 PMC6468084 30291769

[2] S. A. Rasmussen, D. J. Jamieson, J. S. Bresee, Pandemic influenza and pregnant women. Emerg. Infect. Dis. 14, 95–100 (2008). 10.3201/eid1401.070667 PMC2600164 18258087

[3] S. Chandra, J. Christensen, S. E. Mamelund, N. Paneth, Short-term birth sequelae of the 1918-1920 influenza pandemic in the United States: State-level analysis. Am. J. Epidemiol. 187, 2585–2595 (2018). 10.1093/aje/kwy153 PMC7314232 30059982

[4] H. Nishiura, Excess risk of stillbirth during the 1918-1920 influenza pandemic in Japan. Eur. J. Obstet. Gynecol. Reprod. Biol. 147, 115 (2009). 10.1016/j.ejogrb.2009.07.009 19679387

[5] J. M. Hardy, E. N. Azarowicz, A. Mannini, D. N. Medearis Jr., R. E. Cooke, The effect of Asian influenza on the outcome of pregnancy, Baltimore, 1957-1958. Am. J. Public Health Nations Health 51, 1182–1188 (1961). 10.2105/ajph.51.8.1182 PMC1522235 13711529

[6] A. Reid, The effects of the 1918-1919 influenza pandemic on infant and child health in Derbyshire. Med. Hist. 49, 29–54 (2005). 10.1017/s0025727300008279 PMC1088249 15730129

[7] D. W. Freeman, A. Barno, Deaths from Asian influenza associated with pregnancy. Am. J. Obstet. Gynecol. 78, 1172–1175 (1959). 10.1016/0002-9378(59)90570-8 13824729

[8] M. Greenberg, H. Jacobziner, J. Pakter, B. A. Weisl, Maternal mortality in the epidemic of Asian influenza, New York City, 1957. Am. J. Obstet. Gynecol. 76, 897–902 (1958). 10.1016/0002-9378(58)90027-9 13583035

[9] M. Pierce, J. J. Kurinczuk, P. Spark, P. Brocklehurst, M. Knight, on behalf of UKOSS, Perinatal outcomes after maternal 2009/H1N1 infection: National cohort study. BMJ 342, d3214 (2011). 10.1136/bmj.d3214 PMC3114455 21672992

[10] Centers for Disease Control and Prevention (CDC), Maternal and infant outcomes among severely ill pregnant and postpartum women with 2009 pandemic influenza A (H1N1)--United States, April 2009–August 2010. MMWR Morb. Mortal. Wkly Rep. 60, 1193–1196 (2011). 21900872

[11] S. E. Håberg, L. Trogstad, N. Gunnes, A. J. Wilcox, H. K. Gjessing, S. O. Samuelsen, A. Skrondal, I. Cappelen, A. Engeland, P. Aavitsland, S. Madsen, I. Buajordet, K. Furu, P. Nafstad, S. E. Vollset, B. Feiring, H. Nokleby, P. Magnus, C. Stoltenberg, Risk of fetal death after pandemic influenza virus infection or vaccination. N. Engl. J. Med. 368, 333–340 (2013). 10.1056/NEJMoa1207210 PMC3602844 23323868

[12] S. A. Rasmussen, D. J. Jamieson, T. M. Uyeki, Effects of influenza on pregnant women and infants. Am. J. Obstet. Gynecol. 207, S3–S8 (2012). 10.1016/j.ajog.2012.06.068 22920056

[13] G. W. Kosmak, The occurrence of epidemic influenza in pregnancy. Am. J. Obstet. Gynecol. 239–251 (1919).

[14] J. W. Harris, Influenza occurring in pregnant women: A statistical study of thirteen hundred and fifty cases. JAMA 72, 978–980 (1919).

[15] B. J. S. Al-Haddad, E. Oler, B. Armistead, N. A. Elsayed, D. R. Weinberger, R. Bernier, I. Burd, R. Kapur, B. Jacobsson, C. Wang, I. Mysorekar, L. Rajagopal, K. M. Adams Waldorf, The fetal origins of mental illness. Am. J. Obstet. Gynecol. 221, 549–562 (2019). 10.1016/j.ajog.2019.06.013 PMC6889013 31207234

[16] C. E. Barr, S. A. Mednick, P. Munk-Jorgensen, Exposure to influenza epidemics during gestation and adult schizophrenia. Arch. Gen. Psychiatry 47, 869–874 (1990). 10.1001/archpsyc.1990.01810210077012 2393346

[17] S. E. Canetta, Y. Bao, M. D. T. Co, F. A. Ennis, J. Cruz, M. Terajima, L. Shen, C. Kellendonk, C. A. Schaefer, A. S. Brown, Serological documentation of maternal influenza exposure and bipolar disorder in adult offspring. Am. J. Psychiatry 171, 557–563 (2014). 10.1176/appi.ajp.2013.13070943 PMC4025955 24480930

[18] R. A. Machon, M. O. Huttunen, S. A. Mednick, J. Sinivuo, A. Tanskanen, J. Bunn Watson, M. Henriksson, R. Pyhala, Adult schizotypal personality characteristics and prenatal influenza in a Finnish birth cohort. Schizophr. Res. 54, 7–16 (2002). 10.1016/s0920-9964(01)00346-2 11853973

[19] R. A. Machon, S. A. Mednick, M. O. Huttunen, Adult major affective disorder after prenatal exposure to an influenza epidemic. Arch. Gen. Psychiatry 54, 322–328 (1997). 10.1001/archpsyc.1997.01830160040006 9107148

[20] S. A. Mednick, M. O. Huttunen, R. A. Machón, Prenatal influenza infections and adult schizophrenia. Schizophr. Bull. 20, 263–267 (1994). 10.1093/schbul/20.2.263 8085130

[21] S. A. Mednick, R. A. Machon, M. O. Huttunen, D. Bonett, Adult schizophrenia following prenatal exposure to an influenza epidemic. Arch. Gen. Psychiatry 45, 189–192 (1988). 10.1001/archpsyc.1988.01800260109013 3337616

[22] B. J. S. Al-Haddad, B. Jacobsson, S. Chabra, D. Modzelewska, E. M. Olson, R. Bernier, D. A. Enquobahrie, H. Hagberg, S. Östling, L. Rajagopal, K. M. Adams Waldorf, V. Sengpiel, Long-term risk of neuropsychiatric disease after exposure to infection in utero. JAMA Psychiatry 76, 594–602 (2019). 10.1001/jamapsychiatry.2019.0029 PMC6551852 30840048

[23] S. Liong, O. Oseghale, E. E. To, K. Brassington, J. R. Erlich, R. Luong, F. Liong, R. Brooks, C. Martin, S. O’Toole, A. Vinh, L. A. J. O’Neill, S. Bozinovski, R. Vlahos, P. C. Papagianis, J. J. O’Leary, D. A. Brooks, S. Selemidis, Influenza A virus causes maternal and fetal pathology via innate and adaptive vascular inflammation in mice. Proc. Natl. Acad. Sci. U.S.A. 117, 24964–24973 (2020). 10.1073/pnas.2006905117 PMC7547222 32958663

[24] H. Van Campen, J. V. Bishop, V. M. Abrahams, H. Bielefeldt-Ohmann, C. K. Mathiason, G. J. Bouma, Q. A. Winger, C. E. Mayo, R. A. Bowen, T. R. Hansen, Maternal influenza A virus infection restricts fetal and placental growth and adversely affects the fetal thymic transcriptome. Viruses 12, 1003 (2020). 10.3390/v12091003 PMC7551156 32911797

[25] D. Swieboda, E. Q. Littauer, J. T. Beaver, L. K. Mills, K. M. Bricker, E. S. Esser, O. Q. Antao, D. T. Williams, I. Skountzou, Pregnancy downregulates plasmablast metabolic gene expression following influenza without altering long-term antibody function. Front. Immunol. 11, 1785 (2020). 10.3389/fimmu.2020.01785 PMC7457062 32922392

[26] E. Q. Littauer, E. S. Esser, O. Q. Antao, E. V. Vassilieva, R. W. Compans, I. Skountzou, H1N1 influenza virus infection results in adverse pregnancy outcomes by disrupting tissue-specific hormonal regulation. PLOS Pathog. 13, e1006757 (2017). 10.1371/journal.ppat.1006757 PMC5720832 29176767

[27] P. S. Creisher, S. L. Klein, Pathogenesis of viral infections during pregnancy. Clin. Microbiol. Rev. 37, e0007323 (2024). 10.1128/cmr.00073-23 PMC11237665 38421182

[28] P. S. Creisher, M. A. Parish, J. Lei, J. Liu, J. L. Perry, A. D. Campbell, M. L. Sherer, I. Burd, S. L. Klein, Suppression of progesterone by influenza A virus mediates adverse maternal and fetal outcomes in mice. mBio 15, e0306523 (2024). 10.1128/mbio.03065-23 PMC10865978 38190129

[29] M. Li, A. Brokaw, A. M. Furuta, B. Coler, V. Obregon-Perko, A. Chahroudi, H.-Y. Wang, S. R. Permar, C. E. Hotchkiss, T. G. Golos, L. Rajagopal, K. M. Adams Waldorf, Non-human primate models to investigate mechanisms of infection-associated fetal and pediatric injury, teratogenesis and stillbirth. Front. Genet. 12, 680342 (2021). 10.3389/fgene.2021.680342 PMC8287178 34290739

[30] K. M. Adams Waldorf, C. E. Rubens, M. G. Gravett, Use of nonhuman primate models to investigate mechanisms of infection-associated preterm birth. BJOG 118, 136–144 (2011). 10.1111/j.1471-0528.2010.02728.x PMC3060081 21040390

[31] N. M. Bouvier, A. C. Lowen, Animal models for influenza virus pathogenesis and transmission. Viruses 2, 1530–1563 (2010). 10.3390/v20801530 PMC3063653 21442033

[32] M. T. Koday, J. A. Leonard, P. Munson, A. Forero, M. Koday, D. L. Bratt, J. T. Fuller, R. Murnane, S. Qin, T. A. Reinhart, K. Duus, I. Messaoudi, A. L. Hartman, K. Stefano-Cole, J. Morrison, M. G. Katze, D. H. Fuller, Multigenic DNA vaccine induces protective cross-reactive T cell responses against heterologous influenza virus in nonhuman primates. PLOS ONE 12, e0189780 (2017). 10.1371/journal.pone.0189780 PMC5739435 29267331

[33] S. J. Short, G. R. Lubach, A. I. Karasin, C. W. Olsen, M. Styner, R. C. Knickmeyer, J. H. Gilmore, C. L. Coe, Maternal influenza infection during pregnancy impacts postnatal brain development in the rhesus monkey. Biol. Psychiatry 67, 965–973 (2010). 10.1016/j.biopsych.2009.11.026 PMC3235476 20079486

[34] A. E. Mayer, G. D. Parks, An AGM model for changes in complement during pregnancy: Neutralization of influenza virus by serum is diminished in late third trimester. PLOS ONE 9, e112749 (2014). 10.1371/journal.pone.0112749 PMC4237339 25409303

[35] K. M. Adams Waldorf, B. R. Nelson, J. E. Stencel-Baerenwald, C. Studholme, R. P. Kapur, B. Armistead, C. L. Walker, S. Merillat, J. Vornhagen, J. Tisoncik-Go, A. Baldessari, M. Coleman, M. K. Dighe, D. W. W. Shaw, J. A. Roby, V. Santana-Ufret, E. Boldenow, J. Li, X. Gao, M. A. Davis, J. A. Swanstrom, K. Jensen, D. G. Widman, R. S. Baric, J. T. Medwid, K. A. Hanley, J. Ogle, G. M. Gough, W. Lee, C. English, W. M. Durning, J. Thiel, C. Gatenby, E. C. Dewey, M. R. Fairgrieve, R. D. Hodge, R. F. Grant, L. Kuller, W. B. Dobyns, R. F. Hevner, M. Gale Jr., L. Rajagopal, Congenital Zika virus infection as a silent pathology with loss of neurogenic output in the fetal brain. Nat. Med. 24, 368–374 (2018). 10.1038/nm.4485 PMC5839998 29400709

[36] E. Boldenow, C. Gendrin, L. Ngo, C. Bierle, J. Vornhagen, M. Coleman, S. Merillat, B. Armistead, C. Whidbey, V. Alishetti, V. Santana-Ufret, J. Ogle, M. Gough, S. Srinouanprachanh, J. W. MacDonald, T. K. Bammler, A. Bansal, H. D. Liggitt, L. Rajagopal, K. M. Adams Waldorf, Group B *Streptococcus* circumvents neutrophils and neutrophil extracellular traps during amniotic cavity invasion and preterm labor. Sci. Immunol. 1, eaah4576 (2016). 10.1126/sciimmunol.aah4576 PMC5089172 27819066

[37] J. M. Cline, D. Dixon, J. Ernerudh, M. M. Faas, C. Göhner, J.-D. Häger, U. R. Markert, C. Pfarrer, J. Svensson-Arvelund, E. Buse, The placenta in toxicology. Part III: Pathologic assessment of the placenta. Toxicol. Pathol. 42, 339–344 (2014). 10.1177/0192623313482207 23531795

[38] T. E. Bunton, Incidental lesions in nonhuman primate placentae. Vet. Pathol. 23, 431–438 (1986). 10.1177/030098588602300413 3750737

[39] E. P. C. T. de Rijk, E. Van Esch, The macaque placenta—A mini-review. Toxicol. Pathol. 36, 108S–118S (2008).

[40] J. D. Matute, B. Finander, D. Pepin, X. Ai, N. P. Smith, J. Z. Li, A. G. Edlow, A. C. Villani, P. H. Lerou, B. T. Kalish, Single-cell immunophenotyping of the fetal immune response to maternal SARS-CoV-2 infection in late gestation. Pediatr. Res. 91, 1090–1098 (2022). 10.1038/s41390-021-01793-z PMC8573077 34750520

[41] B. Coler, K. Adams Waldorf, Maternal COVID-19 leaves a lasting immunological impression on the fetus. Nat. Immunol. 22, 1472–1473 (2021). 10.1038/s41590-021-01072-3 34782774

[42] C. Coler, E. King-Nakaoka, E. Every, S. Chima, A. Vong, B. Del Rosario, R. VanAbel, K. M. Adams Waldorf, Impact of infections during pregnancy on transplacental antibody transfer. Vaccine 12, 1199 (2024). 10.3390/vaccines12101199 PMC11512415 39460363

[43] E. González-Mesa, E. García-Fuentes, R. Carvia-Pontiasec, A. I. Lavado-Fernández, C. Cuenca-Marín, M. Suárez-Arana, M. Blasco-Alonso, B. Benítez-Lara, L. Mozas-Benítez, A. González-Cazorla, H. Egeberg-Neverdal, J. S. Jiménez-López, Transmitted fetal immune response in cases of SARS-CoV-2 infections during pregnancy. Diagnostics 12, 245 (2022). 10.3390/diagnostics12020245 PMC8870756 35204335

[44] S. Gee, M. Chandiramani, J. Seow, E. Pollock, C. Modestini, A. Das, T. Tree, K. J. Doores, R. M. Tribe, D. L. Gibbons, The legacy of maternal SARS-CoV-2 infection on the immunology of the neonate. Nat. Immunol. 22, 1490–1502 (2021). 10.1038/s41590-021-01049-2 34616036

[45] H. Jacobsen, K. Walendy-Gnirss, N. Tekin-Bubenheim, N. M. Kouassi, I. Ben-Batalla, N. Berenbrok, M. Wolff, V. P. Dos Reis, M. Zickler, L. Scholl, A. Gries, H. Jania, A. Kloetgen, A. Dusedau, G. Pilnitz-Stolze, A. Jeridi, A. O. Yildirim, H. Fuchs, V. Gailus-Durner, C. Stoeger, M. H. de Angelis, T. Manuylova, K. Klingel, F. J. Culley, J. Behrends, S. Loges, B. Schneider, S. Krauss-Etschmann, P. Openshaw, G. Gabriel, Offspring born to influenza A virus infected pregnant mice have increased susceptibility to viral and bacterial infections in early life. Nat. Commun. 12, 4957 (2021). 10.1038/s41467-021-25220-3 PMC8368105 34400653

[46] S. Liong, M. A. Miles, M. Mohsenipour, F. Liong, E. L. Hill-Yardin, S. Selemidis, Influenza A virus infection during pregnancy causes immunological changes in gut-associated lymphoid tissues of offspring mice. Am. J. Physiol. Gastrointest. Liver Physiol. 325, G230–G238 (2023). 10.1152/ajpgi.00062.2023 PMC10435073 37431584

[47] C. Pfeifer, M. J. Bunders, Maternal HIV infection alters the immune balance in the mother and fetus; implications for pregnancy outcome and infant health. Curr. Opin. HIV AIDS 11, 138–145 (2016). 10.1097/COH.0000000000000239 26679415

[48] M. Clerici, M. Saresella, F. Colombo, S. Fossati, N. Sala, D. Bricalli, M. L. Villa, P. Ferrante, L. Dally, A. Vigano, T-lymphocyte maturation abnormalities in uninfected newborns and children with vertical exposure to HIV. Blood 96, 3866–3871 (2000). 11090071

[49] K. C. Rich, J. N. Siegel, C. Jennings, R. J. Rydman, A. L. Landay, Function and phenotype of immature CD4^+^ lymphocytes in healthy infants and early lymphocyte activation in uninfected infants of human immunodeficiency virus-infected mothers. Clin. Diagn. Lab. Immunol. 4, 358–361 (1997). 10.1128/cdli.4.3.358-361.1997 PMC170532 9144377

[50] E. Ono, A. M. Nunes dos Santos, R. C. de Menezes Succi, D. M. Machado, D. S. A. de Angelis, R. Salomão, E. G. Kallas, M. I. de Moraes-Pinto, Imbalance of naive and memory T lymphocytes with sustained high cellular activation during the first year of life from uninfected children born to HIV-1-infected mothers on HAART. Braz. J. Med. Biol. Res. 41, 700–708 (2008). 10.1590/s0100-879x2008000800011 18797705

[51] A. S. Brown, S. Vinogradov, W. S. Kremen, J. H. Poole, R. F. Deicken, J. D. Penner, I. W. McKeague, A. Kochetkova, D. Kern, C. A. Schaefer, Prenatal exposure to maternal infection and executive dysfunction in adult schizophrenia. Am. J. Psychiatry 166, 683–690 (2009). 10.1176/appi.ajp.2008.08010089 PMC2885160 19369317

[52] G. M. Khandaker, J. Zimbron, G. Lewis, P. B. Jones, Prenatal maternal infection, neurodevelopment and adult schizophrenia: A systematic review of population-based studies. Psychol. Med. 43, 239–257 (2013). 10.1017/S0033291712000736 PMC3479084 22717193

[53] S. Davidson, M. K. Maini, A. Wack, Disease-promoting effects of type I interferons in viral, bacterial, and coinfections. J. Interferon Cytokine Res. 35, 252–264 (2015). 10.1089/jir.2014.0227 PMC4389918 25714109

[54] L. J. Yockey, K. A. Jurado, N. Arora, A. Millet, T. Rakib, K. M. Milano, A. K. Hastings, E. Fikrig, Y. Kong, T. L. Horvath, S. Weatherbee, H. J. Kliman, C. B. Coyne, A. Iwasaki, Type I interferons instigate fetal demise after Zika virus infection. Sci. Immunol. 3, eaao1680 (2018). 10.1126/sciimmunol.aao1680 PMC6049088 29305462

[55] F. M. Szaba, M. Tighe, L. W. Kummer, K. G. Lanzer, J. M. Ward, P. Lanthier, I. J. Kim, A. Kuki, M. A. Blackman, S. J. Thomas, J. S. Lin, Zika virus infection in immunocompetent pregnant mice causes fetal damage and placental pathology in the absence of fetal infection. PLOS Pathog. 14, e1006994 (2018). 10.1371/journal.ppat.1006994 PMC5909921 29634758

[56] F. McNab, K. Mayer-Barber, A. Sher, A. Wack, A. O’Garra, Type I interferons in infectious disease. Nat. Rev. Immunol. 15, 87–103 (2015). 10.1038/nri3787 PMC7162685 25614319

[57] A. M. Siston, S. A. Rasmussen, M. A. Honein, A. M. Fry, K. Seib, W. M. Callaghan, J. Louie, T. J. Doyle, M. Crockett, R. Lynfield, Z. Moore, C. Wiedeman, M. Anand, L. Tabony, C. F. Nielsen, K. Waller, S. Page, J. M. Thompson, C. Avery, C. B. Springs, T. Jones, J. L. Williams, K. Newsome, L. Finelli, D. J. Jamieson, Pandemic H1N1 Influenza in Pregnancy Working Group, Pandemic 2009 influenza A(H1N1) virus illness among pregnant women in the United States. JAMA 303, 1517–1525 (2010). 10.1001/jama.2010.479 PMC5823273 20407061

[58] K. M. Neuzil, G. W. Reed, E. F. Mitchel, L. Simonsen, M. R. Griffin, Impact of influenza on acute cardiopulmonary hospitalizations in pregnant women. Am. J. Epidemiol. 148, 1094–1102 (1998). 10.1093/oxfordjournals.aje.a009587 9850132

[59] ACOG Committee Statement No. 7: Influenza in Pregnancy: Prevention and Treatment. Obstet. Gynecol. 143, e24–e30 (2024). 10.1097/AOG.0000000000005479 38016152

[60] H. Razzaghi, K. E. Kahn, K. Calhoun, E. Garacci, T. H. Skoff, S. R. Ellington, T. C. Jatlaoui, C. L. Black, Influenza, Tdap, and COVID-19 vaccination coverage and hesitancy among pregnant women—United States, April 2023. MMWR Morb. Mortal. Wkly Rep. 72, 1065–1071 (2023). 10.15585/mmwr.mm7239a4 PMC10545430 37768879

[61] A. K. Regan, H. C. Moore, N. de Klerk, S. B. Omer, G. Shellam, D. B. Mak, P. V. Effler, Seasonal trivalent influenza vaccination during pregnancy and the incidence of stillbirth: Population-based retrospective cohort study. Clin. Infect. Dis. 62, 1221–1227 (2016). 10.1093/cid/ciw082 27033634

[62] M. Sarna, C. C. Blyth, H. C. Moore, G. Pereira, L. McHugh, M. Binks, K. Lust, P. Van Buynder, D. Foo, R. Andrews, A. K. Regan, Neonatal and infant mortality after maternal influenza and pertussis vaccination: Probabilistically linked cohort study. Hum. Vaccin. Immunother. 21, 2587307 (2025). 10.1080/21645515.2025.2587307 PMC12645885 41263480

[63] K. N. Bratton, M. T. Wardle, W. A. Orenstein, S. B. Omer, Maternal Influenza Immunization and Birth Outcomes of Stillbirth and Spontaneous Abortion: A Systematic Review and Meta-analysis. Clin. Infect. Dis. 60, e11–e19 (2014). 10.1093/cid/ciu915 25409473

[64] K. Zaman, E. Roy, S. E. Arifeen, M. Rahman, R. Raqib, E. Wilson, S. B. Omer, N. S. Shahid, R. F. Breiman, M. C. Steinhoff, Effectiveness of maternal influenza immunization in mothers and infants. N. Engl. J. Med. 359, 1555–1564 (2008). 10.1056/NEJMoa0708630 18799552

[65] M. G. Thompson, D.-K. Li, P. Shifflett, L. Z. Sokolow, J. R. Ferber, S. Kurosky, S. Bozeman, S. B. Reynolds, R. Odouli, M. L. Henninger, T. L. Kauffman, L. A. Avalos, S. Ball, J. L. Williams, S. A. Irving, D. K. Shay, A. L. Naleway, Pregnancy and Influenza Project Workgroup, Effectiveness of Seasonal Trivalent Influenza Vaccine for Preventing Influenza Virus Illness Among Pregnant Women : A Population-Based Case-Control Study During the 2010–2011 and 2011–2012 Influenza Seasons. Clin. Infect. Dis. 58, 449–457 (2013). 10.1093/cid/cit750 24280090

[66] Y. Itoh, K. Shinya, M. Kiso, T. Watanabe, Y. Sakoda, M. Hatta, Y. Muramoto, D. Tamura, Y. Sakai-Tagawa, T. Noda, S. Sakabe, M. Imai, Y. Hatta, S. Watanabe, C. Li, S. Yamada, K. Fujii, S. Murakami, H. Imai, S. Kakugawa, M. Ito, R. Takano, K. Iwatsuki-Horimoto, M. Shimojima, T. Horimoto, H. Goto, K. Takahashi, A. Makino, H. Ishigaki, M. Nakayama, M. Okamatsu, K. Takahashi, D. Warshauer, P. A. Shult, R. Saito, H. Suzuki, Y. Furuta, M. Yamashita, K. Mitamura, K. Nakano, M. Nakamura, R. Brockman-Schneider, H. Mitamura, M. Yamazaki, N. Sugaya, M. Suresh, M. Ozawa, G. Neumann, J. Gern, H. Kida, K. Ogasawara, Y. Kawaoka, In vitro and in vivo characterization of new swine-origin H1N1 influenza viruses. Nature 460, 1021–1025 (2009). 10.1038/nature08260 PMC2748827 19672242

[67] J. T. Go, S. E. Belisle, N. Tchitchek, T. M. Tumpey, W. Ma, J. A. Richt, D. Safronetz, H. Feldmann, M. G. Katze, 2009 pandemic H1N1 influenza virus elicits similar clinical course but differential host transcriptional response in mouse, macaque, and swine infection models. BMC Genomics 13, 627 (2012). 10.1186/1471-2164-13-627 PMC3532173 23153050

[68] P. Mooij, G. Koopman, D. Mortier, M. van Heteren, H. Oostermeijer, Z. Fagrouch, R. de Laat, G. Kobinger, Y. Li, E. J. Remarque, I. Kondova, E. J. Verschoor, W. M. J. M. Bogers, Pandemic swine-origin H1N1 influenza virus replicates to higher levels and induces more fever and acute inflammatory cytokines in cynomolgus versus rhesus monkeys and can replicate in common marmosets. PLOS ONE 10, e0126132 (2015). 10.1371/journal.pone.0126132 PMC4422689 25946071

[69] L. J. Reed, H. Muench, A simple method of estimating fifty percent endpoints. Am. J. Epidemiol. 27, 493–497 (1938).

[70] Y. Yang, F. Huang, R. Gonzalez, W. Wang, G. Lu, Y. Li, G. Vernet, Q. Jin, J. Wang, Evaluation of twelve real-time reverse transcriptase PCR primer-probe sets for detection of pandemic influenza A/H1N1 2009 virus. J. Clin. Microbiol. 49, 1434–1440 (2011). 10.1128/JCM.01914-10 PMC3122877 21289144

[71] M. A. O’Connor, J. Tisoncik-Go, T. B. Lewis, C. J. Miller, D. Bratt, C. R. Moats, P. T. Edlefsen, J. Smedley, N. R. Klatt, M. Gale Jr., D. H. Fuller, Early cellular innate immune responses drive Zika viral persistence and tissue tropism in pigtail macaques. Nat. Commun. 9, 3371 (2018). 10.1038/s41467-018-05826-w PMC6105614 30135445

[72] M. D. Robinson, D. J. McCarthy, G. K. Smyth, edgeR: A Bioconductor package for differential expression analysis of digital gene expression data. Bioinformatics 26, 139–140 (2010). 10.1093/bioinformatics/btp616 PMC2796818 19910308

[73] C. W. Law, Y. Chen, W. Shi, G. K. Smyth, voom: Precision weights unlock linear model analysis tools for RNA-seq read counts. Genome Biol. 15, R29 (2014). 10.1186/gb-2014-15-2-r29 PMC4053721 24485249

[74] A. Subramanian, P. Tamayo, V. K. Mootha, S. Mukherjee, B. L. Ebert, M. A. Gillette, A. Paulovich, S. L. Pomeroy, T. R. Golub, E. S. Lander, J. P. Mesirov, Gene set enrichment analysis: A knowledge-based approach for interpreting genome-wide expression profiles. Proc. Natl. Acad. Sci. U.S.A. 102, 15545–15550 (2005). 10.1073/pnas.0506580102 PMC1239896 16199517

[75] Z. Gu, R. Eils, M. Schlesner, Complex heatmaps reveal patterns and correlations in multidimensional genomic data. Bioinformatics 32, 2847–2849 (2016). 10.1093/bioinformatics/btw313 27207943

[76] P. J. Aphalo, *Learn R: As a Language* (The R Series, Chapman and Hall/CRC Press, ed. 2, 2024).

