# Supplementary material for: Influenza A virus infection induces immune dysregulation in the placenta and fetus without vertical transmission in nonhuman primates
Source: Sci Adv. 2026 Oct 9;12(41):eaee9861. doi: 10.1126/sciadv.aee9861 (PMC13651839; doi:10.1126/sciadv.aee9861)
Supplement: Supplementary file 1 [file sciadv.aee9861_sm.pdf]

Science Advances
AAAS

Supplementary Materials for

Influenza A virus infection induces immune dysregulation in the placenta and fetus without vertical transmission in nonhuman primates

Orlando Cervantes *et al.*

Corresponding author: Kristina M. Adams Waldorf, adamsk@uw.edu

Sci. Adv. **12**, eaee9861 (2026)
DOI: 10.1126/sciadv.aee9861

This PDF file includes:

Tables S1 to S5
Figs. S1 to S20

Table S1. Animal Demographics.

ID	Inoculum	Inoculation to Delivery Interval (Days)	Dam Age at Necropsy (Years)	Fetal Age at Inoculation (Days)	Fetal Age at Delivery (Days)	Fetal Sex	Fetal Weight(g)
CTRL1	Media	100	14.0	59	160	F	431.9
CTRL2	Media	23	13.5	132	155	M	573
CTRL3	Media	58	7.4	99	157 -	F	351
CTRL4	Media	92	12.0	64	155	F	467.2
CTRL5	Media	20	5.2	138	158	F	499.2
CTRL6	Media	21	9.7	133	154	F	411.9
CTRL7	Media	20	10.8	127	147	M	469
CTRL8	Media	3	12.9	141	144	M	412.6
CTRL9	Media	3	5.3	145	148	F	340.3
CTRL10	Saline	7	10.1	136	143	M	405
CTRL11	Saline	7	5.6	138	145	M	316
CTRL12	Saline	7	10.7	132	139	M	340
CTRL13	Saline	1	5.7	127	128	M	242
CTRL14	Saline	1	6.1	133	134	F	264
CTRL15	Saline	1	14.7	135	136	F	299.5
CTRL16	Saline	1	12.7	131	132	F	298.6
FLU1	H1N1	5	12.2	125	130	M	291
FLU2	H1N1	5	12.7	126	131	F	297.2
FLU3	H1N1	5	6.6	127	132	F	258.3
FLU4	H1N1	5	15.5	138	143	F	358
FLU5	H1N1	5	10.3	134	139	F	372.1
FLU6	H1N1	5	7.0	131	135	M	334.2
FLU7	H1N1	5	13.8	129	134	M	318.1
FLU8	H1N1	5	8.5	127	132	M	277.1
FLU9	H1N1	5	7.0	116	121	M	264.1
FLU10	H1N1	5	4.4	126	131	F	316.9
FLU11	H1N1	5	11.8	134	139	M	356.3

This table shows the inoculum, inoculation to delivery interval, fetal age at inoculation and delivery, fetal sex, and fetal weight. Abbreviations: F, female; M, male.

Table S2. Lung and Placental Pathology

ID	Dam	Placenta			
	Maternal Lung Pathology	Maternal Inflammatory Response	Fetal Inflammatory Response	Infarction	Necrotizing Villitis
CTRL1	None	NEG	NEG	NEG	NEG
CTRL2	None	NEG	NEG	NEG	NEG
CTRL3	None	NEG	NEG	NEG	NEG
CTRL4	None	NEG	NEG	NEG	NEG
CTRL5	None	NEG	NEG	**POS**	NEG
CTRL6	None	NEG	NEG	NEG	NEG
CTRL7	None	NEG	NEG	NEG	**POS**
CTRL8	None	NEG	NEG	NEG	**POS**
CTRL9	None	NEG	NEG	NEG	NEG
CTRL10	None	NEG	NEG	NEG	NEG
CTRL11	None	NEG	NEG	NEG	**POS**
CTRL12	None	NEG	NEG	NEG	NEG
CTRL13	None	NEG	NEG	NEG	NEG
CTRL14	None	NEG	NEG	NEG	NEG
CTRL15	None	NEG	NEG	NEG	**POS**
CTRL16	None	NEG	NEG	NEG	NEG
FLU1	Pneumonia, multifocal to coalescing, mild to moderate, mixed, with multifocal pleural fibrosis	NEG	NEG	NEG	NEG
FLU2	Pneumonia, multifocal to diffuse, interstitial, mild to moderate, mixed, with multifocal pleural fibrosis	NEG	NEG	NEG	NEG
FLU3	Pneumonia, interstitial to diffuse, mild (left) to severe (right), with alveolar edema and hyaline membranes	NEG	NEG	**POS**	NEG
FLU4	Mild interstitial pneumonitis, mixed, with mild deep alveolar edema	NEG	NEG	NEG	**POS**
FLU5	Pneumonia, multifocal, moderate, mixed, with	NEG	NEG	**POS**	NEG

	edema and respiratory epithelial hyperplasia				
FLU6	Pneumonia, neutrophilic to mixed, severe, with edema	NEG	NEG	NEG	**POS**
FLU7	Pneumonia, interstitial, mild, chronic to mixed, with peribronchial lymphoid hyperplasia	NEG	NEG	**POS**	NEG
FLU8	Pneumonia, interstitial, mild to severe, neutrophilic to mixed, with alveolar edema and fibrin	NEG	NEG	NEG	**POS**
FLU9	Severe pneumonia with pleural effusion and hilar lymphadenopathy	NEG	NEG	NEG	**POS**
FLU10	Pneumonia, multifocal, mild to moderate, interstitial, neutrophilic to mixed, with alveolar edema	NEG	NEG	NEG	**POS**
FLU11	Severe pneumonia with pleural effusion and hilar lymphadenopathy	NEG	NEG	NEG	NEG

This table shows a summary of maternal lung histopathology and placental pathology. Abbreviations: NEG, negative; POS, positive.

Table S3. IAV H1N1 Viral Testing and Detection in Fetal Tissues

Fetal ID	Tissue								
	Brain	Brainstem	Spinal Cord	Meninges	Heart	Liver	Lung	Spleen	Thymus
FLU1	Neg	Neg	Neg	Neg	Neg	Neg	Neg	Neg	Neg
FLU2	Neg	Neg	Neg	Neg	Neg	Neg	Neg	Neg	Neg
FLU3	Neg	Neg	Neg	NT	Neg	Neg	Neg	Neg	Neg
FLU4	Neg	Neg	Neg	NT	Neg	Neg	Neg	Neg	Neg
FLU5	Neg	Neg	Neg	NT	Neg	Neg	Neg	Neg	Neg
FLU6	Neg	NT	Neg	NT	Neg	Neg	Neg	Neg	Neg
FLU7	Neg	NT	Neg	NT	NT	Neg	Neg	Neg	Neg
FLU8	Neg	NT	Neg	NT	Neg	Neg	Neg	Neg	Neg
FLU9	NT	NT	NT	NT	NT	NT	NT	NT	NT
FLU10	Neg	Neg	Neg	NT	Neg	Neg	Neg	Neg	Neg
FLU11	Neg	Neg	Neg	NT	Neg	Neg	Neg	Neg	Neg

RT-qPCR was performed on samples from different organs of the fetuses from IAV H1N1-infected dams. FLU 9 was stillborn with severe autolysis. RNA quality of the tissue was insufficient for RT-qPCR testing. Abbreviations: NT, not tested; and Neg, negative.

Table S4. Data Subsets to Control for Gestational Age at Inoculation and Delivery

Version	Group	N	Inoculation Gestational Age Mean (Min – Max)	N	Delivery Gestational Age Mean (Min – Max)
Full Sample	FLU	11	128.8 (116.4 - 138.4)	11	133.7 (121.4 – 143.0)
Full Sample	Control	16	123.4 (59.4 - 145.4)	16	146.2 (128.4 - 159.5)
Age Subset	Control	11	133.2 (127.3 - 138.4)	10	139.9 (128.4 - 148.4)

This table shows the inoculation and delivery gestational age mean and range for FLU and Control groups for the full sample (all) versus the gestational age-matched subsets of controls used for cytokines (Fig. S3) and flow cytometry analyses (Fig. S4, S13). Age matching restricted the cohort to animals with an inoculation or delivery gestational age within 7 days of the mean of the FLU cohort.

Table S5. Flow Cytometry Immunophenotyping Gates

Cell Type	Markers
B cells	CD14-/CD3- CD16-/HLA-DR+ CD20+
CD4+ T cells	CD14-/CD16- CD3+/CD4+ CD8-
CD4+CD8+ T cells	CD14-/CD3- CD16+/CD4+CD8+
CD8+ NK cells	CD14-/CD3- CD16+/CD8+ NKG2a+
CD8+ T cells	CD14-/CD16- CD3+/CD4- CD8+
Classical monocytes	CD3- CD20-/ HLA-DR+/ CD16- CD14+
Intermediate monocytes	CD3- CD20-/ HLA-DR+/ CD16+ CD14hi
Myeloid dendritic cells	CD3- CD20-/ HLA-DR+/CD14-/ CD11c+ CD123-
NKT cells	CD14-/CD3+ CD16+
Non-classical monocytes	CD3- CD20-/ HLA-DR+/ CD16+ CD14mid
Plasmacytoid dendritic cells	CD3- CD20-/ HLA-DR+/CD14-/ CD11c- CD123+

Antibody gating used to define immune cell subsets of interest using flow cytometry. All cell populations are live and CD45+ singlets.

Figure S1. Cytokines in Placental Chorionic Villous Tissue

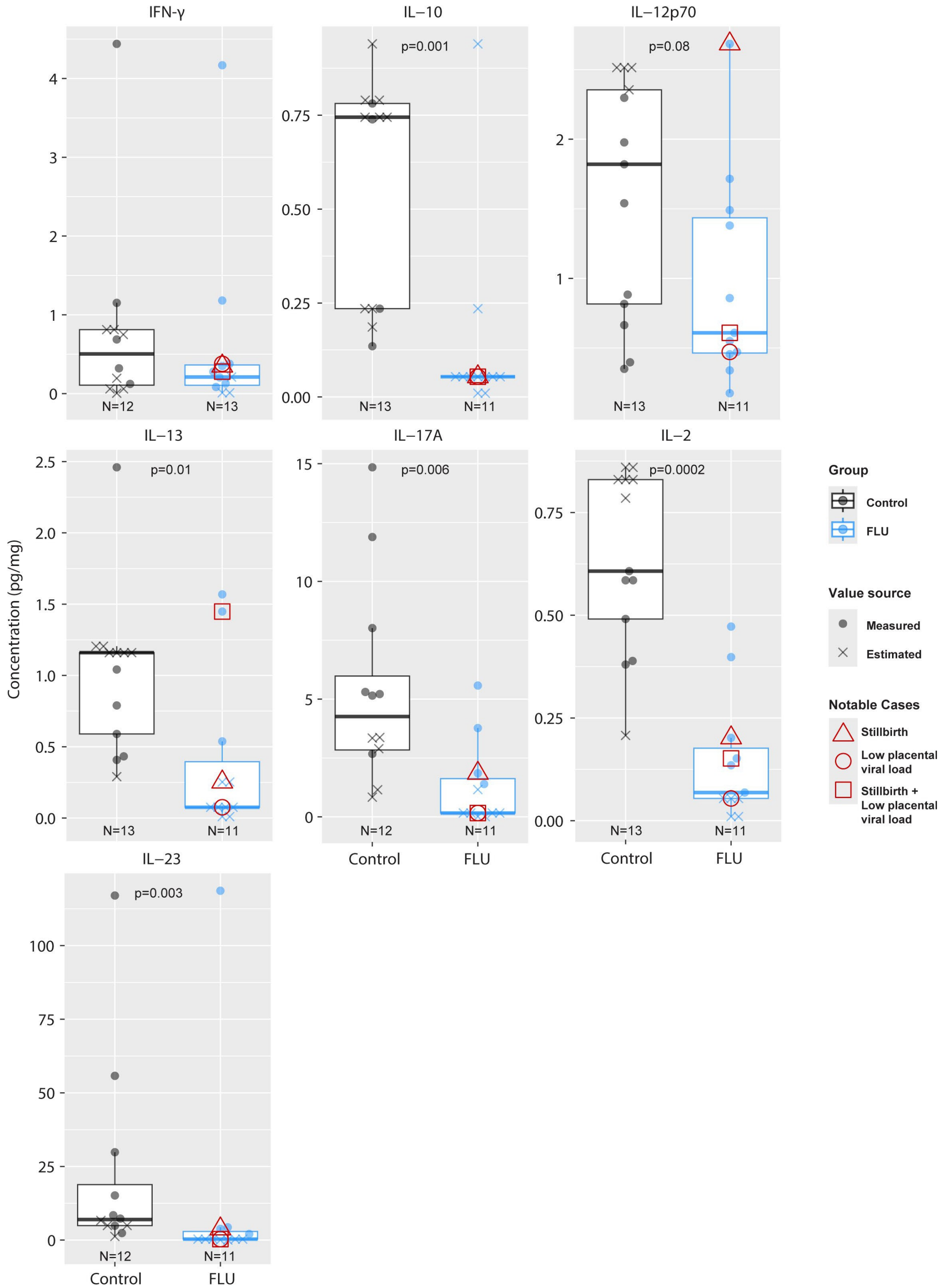

Cytokine concentrations (pg/mg tissue) are shown on the x-axis for placental chorionic villous tissue using a multiplex Luminex assay for the Control and FLU cohorts. Values are shown as solid circles when they were measured within the dynamic range of the Luminex assay and as an 'x' when they were extrapolated from the standard curve. The red circle indicates the cytokine

value from the single animal with a low IAV viral load in the placenta. The red triangles indicate samples obtained from experiments in which there was a stillbirth. The p-values were generated using a Wilcoxon rank-sum test. Abbreviations: IFN-γ, interferon gamma; IL-2, interleukin 2; IL-10, interleukin 10; IL-12p70, interleukin 12; IL-13, interleukin 13; IL-17A, interleukin 17A; and IL-23, interleukin 23. The p-value for CTRL versus FLU cohorts is indicated at the top of each panel if it was less than 0.05. The N is shown at the bottom of each panel.

Figure S2. Cytokines in Amniotic Fluid

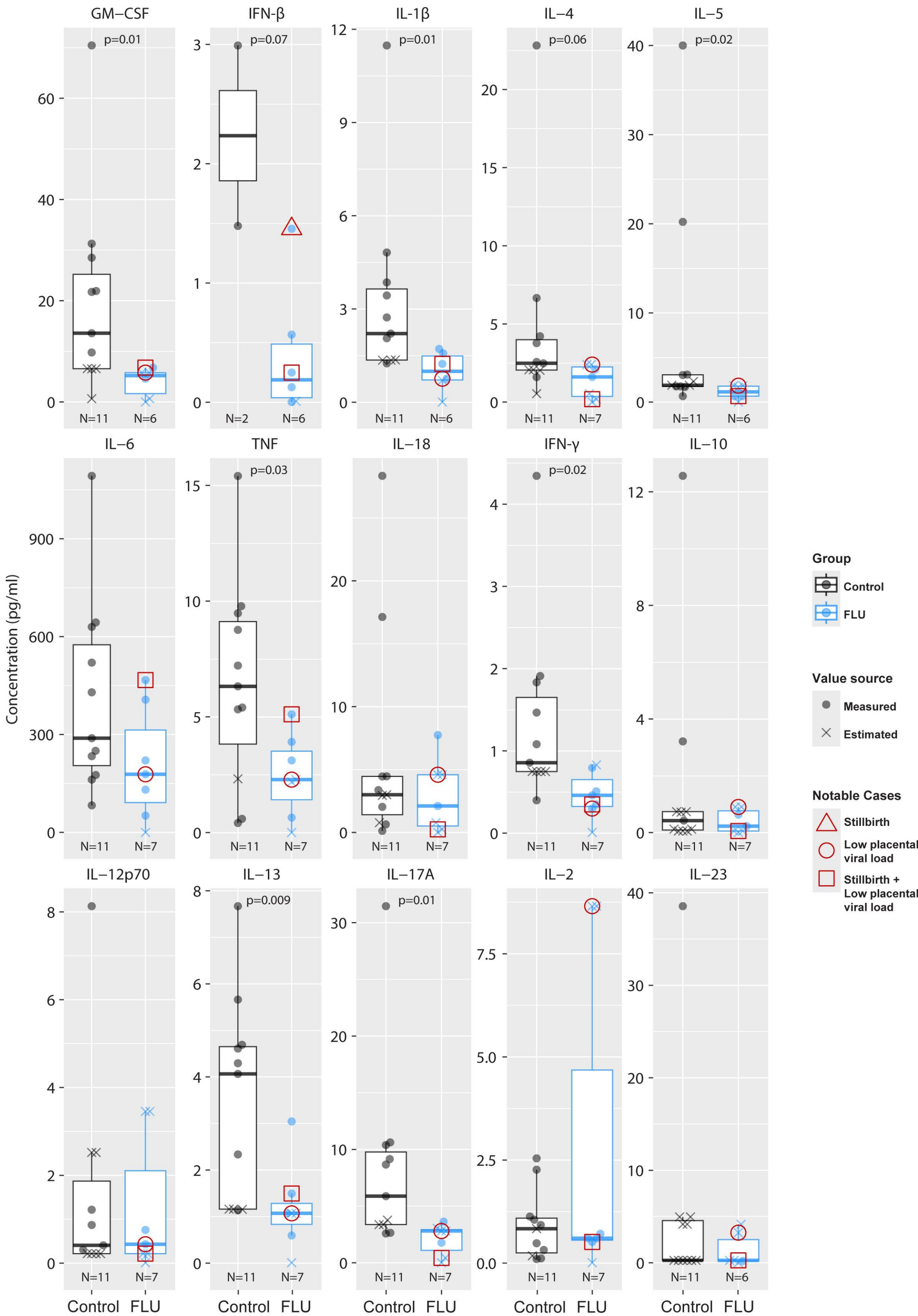

Cytokine concentrations (pg/ml) are shown on the x-axis for amniotic fluid using a multiplex Luminex assay for the Control and FLU cohorts. Values are shown as open circles when they were measured within the working range of the Luminex assay and as an 'x' when they were outside the working range and estimated by extrapolation from the standard curve. The red circle indicates the cytokine value from the single animal with a low IAV viral load in the placenta. The red triangles indicate samples obtained from experiments in which there was a stillbirth. The p-values were generated using a Wilcoxon rank-sum test. The p-value for CTRL vs FLU cohorts is indicated at the top of each panel if it was less than 0.05. The N is shown at the bottom of each panel. Abbreviations: GM-CSF, granulocyte-macrophage colony-stimulating factor; IFN-b, interferon beta; IFN-γ, interferon gamma; IL-1b, interleukin 1 beta; IL-2, interleukin 2; IL-4, interleukin 4; IL-5, interleukin 5; IL-6, interleukin 6; IL-10, interleukin 10; IL-12p70, interleukin 12; IL-13, interleukin 13; IL-17A, interleukin 17A; IL-18, interleukin 18; IL-23, interleukin 23; and TNF, tumor necrosis factor.

Figure S3. Gestational Age Subsetting of Cytokines in the Placenta and Amniotic Fluid

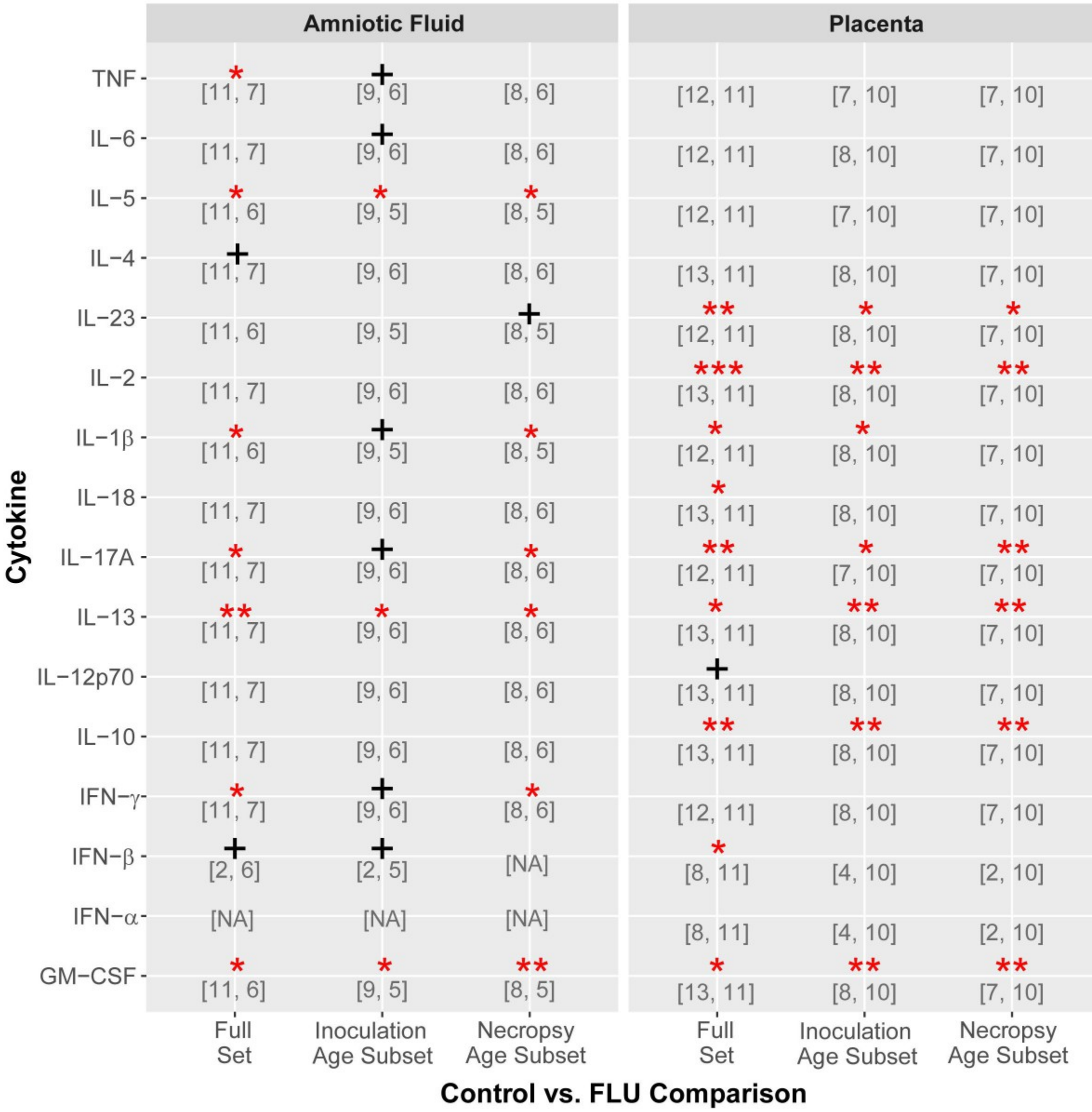

This figure illustrates the significance of the comparisons between CTRL versus FLU animals for a given cytokine (y-axis) in the amniotic fluid (left panel) or placental chorionic villous tissue (right panel). The method of group subsetting is indicated on the x-axis and derives from Table S4. The p-values for each comparison were generated using a Wilcoxon rank-sum test (plus sign: $0.1 < p \leq 0.05$; 1 star: $p<0.05$; 2 stars: $p<0.01$, and 3 stars: $p<0.001$). The sample size for each comparison is shown in brackets, with the left number indicating the CTRL sample size and the right number indicating the FLU sample size.

Figure S4. Gestational Age Subsetting of Placental Immunophenotyping

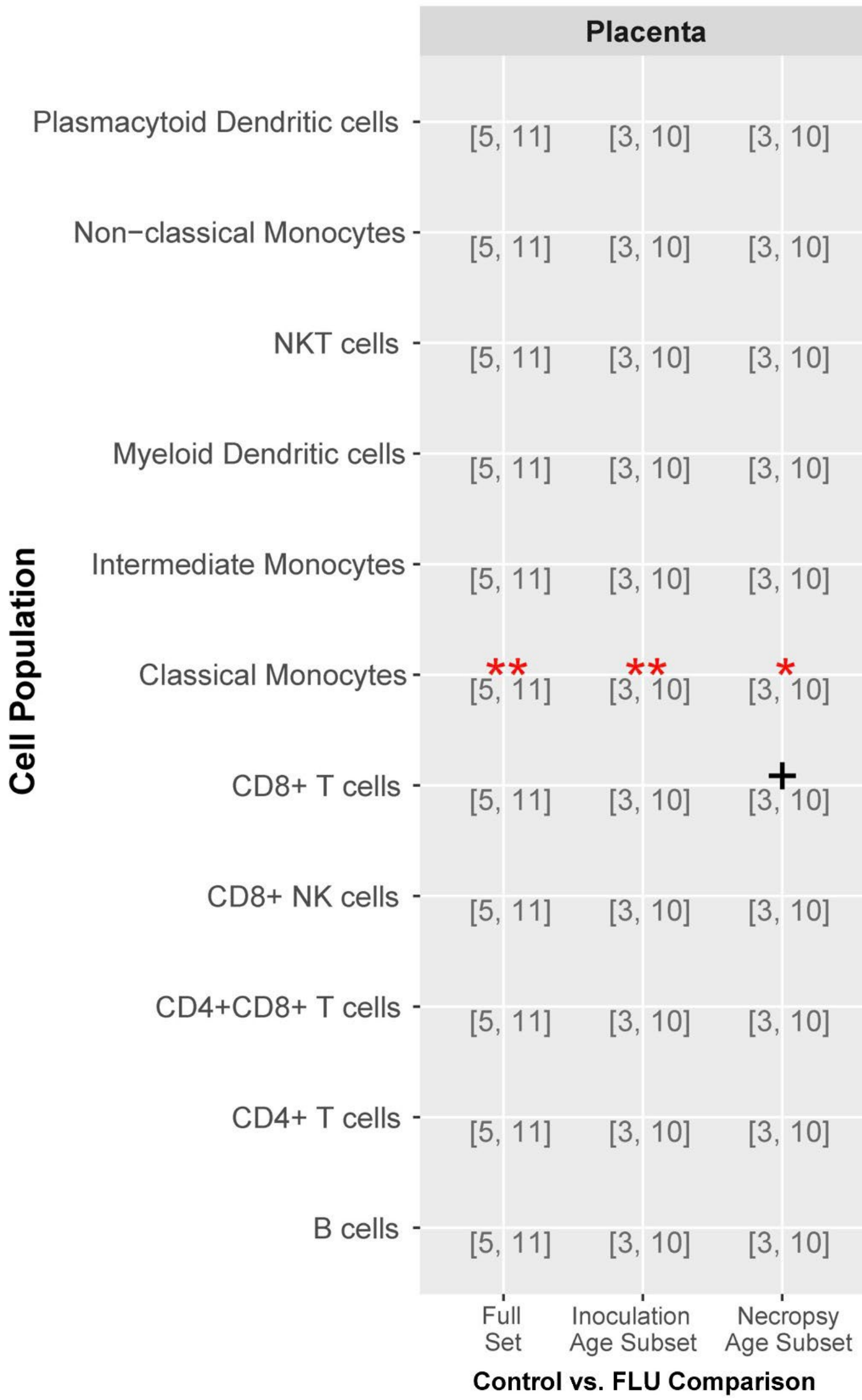

This figure illustrates the significance of the comparisons between CTRL versus FLU animals for a given cell population (y-axis) in the placental chorionic villous tissue. The method of subsetting is indicated on the x-axis and derives from Table S4. The p-values for each comparison were generated using a Wilcoxon rank-sum test (plus sign: $0.1 < p \leq 0.05$;1 star: $p<0.05$; 2 stars: $p<0.01$, 3 stars: $p<0.001$). The sample size for each comparison is shown in brackets, with the left number indicating the CTRL sample size and the right number indicating the FLU sample size.

Figure S5. Multidimensional Scaling of Placental Gene Counts by Total RNA-Seq

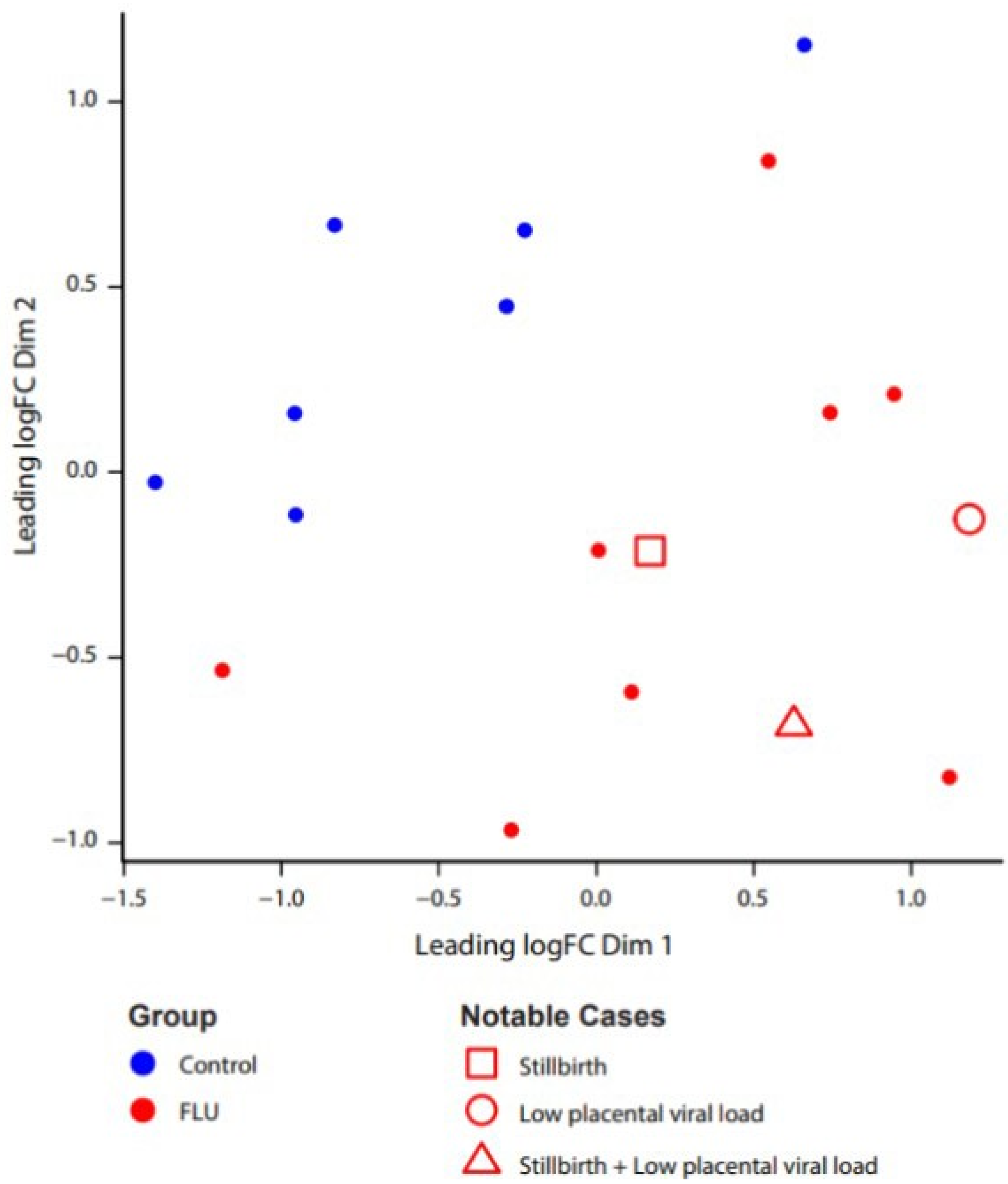

This figure illustrates multidimensional scaling (MDS) of normalized log-transformed gene counts using Euclidean distance to depict sample-to-sample similarity. Each point represents a placenta and is colored by group (FLU: red, N=11; CTRL: blue, N=7). Open red symbols denote FLU samples with low placental IAV vRNA (circle), a stillbirth with undetectable vRNA (square), or a stillbirth with low vRNA (triangle).

Figure S6. Placental nCounter Gene Counts of Differentially Expressed Genes

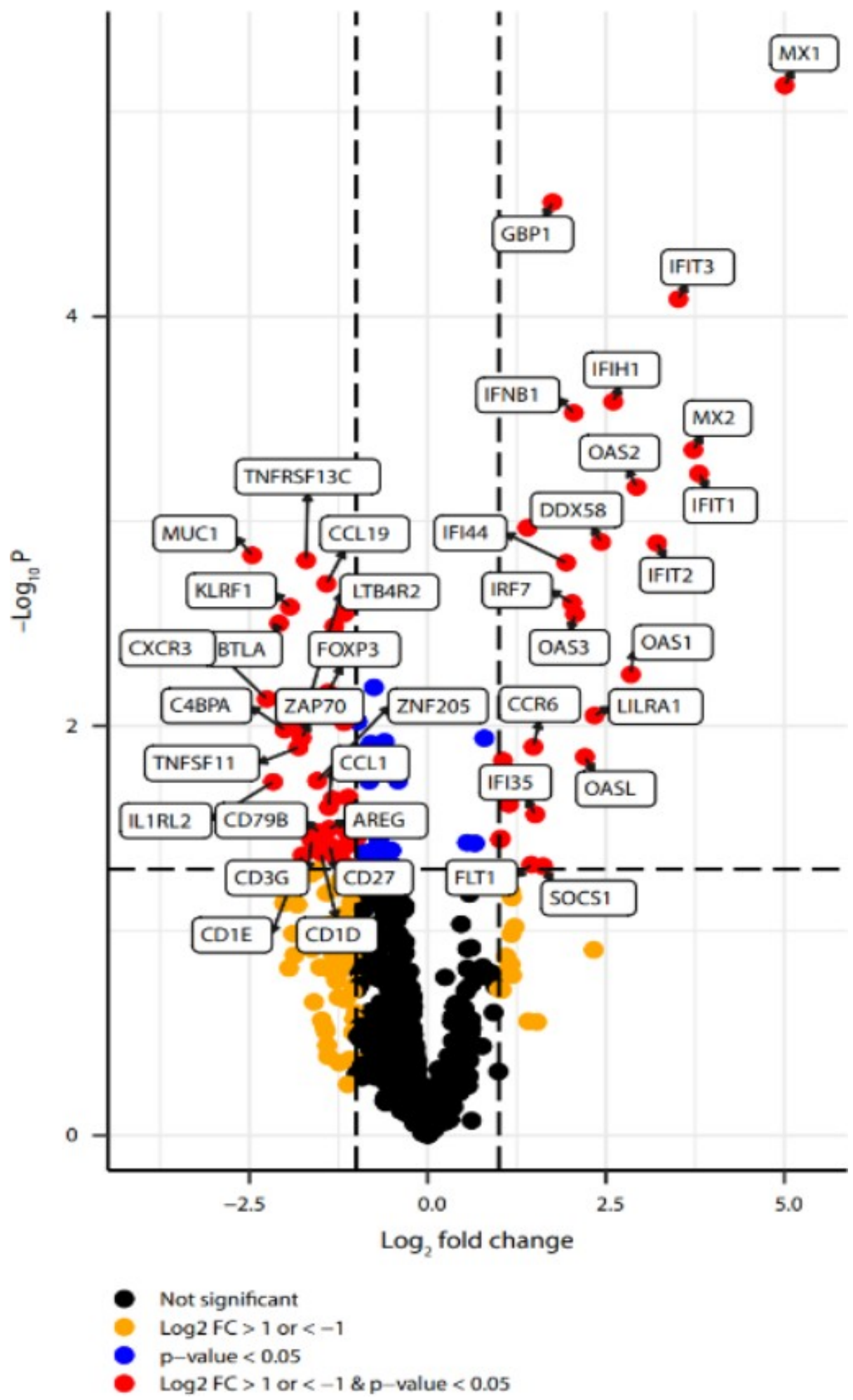

A volcano scatter plot shows the relationship between the log2 fold change and statistical significance (p-value) of differentially expressed genes between the FLU (N=11) and CTRL (N=7) cohorts, as determined by nCounter digital gene counting. Dot colors indicated significance and magnitude: red for genes with a significant |log2 fold change| ≥1; blue for genes with a |log2 fold change| <1 but p <0.05; gold for non-significant genes with a |log2 fold change| ≥1; and black for non-significant genes with a |log2 fold change| <1.

Figure S7. Correlation of nCounter and Total RNA-Seq Gene Counts

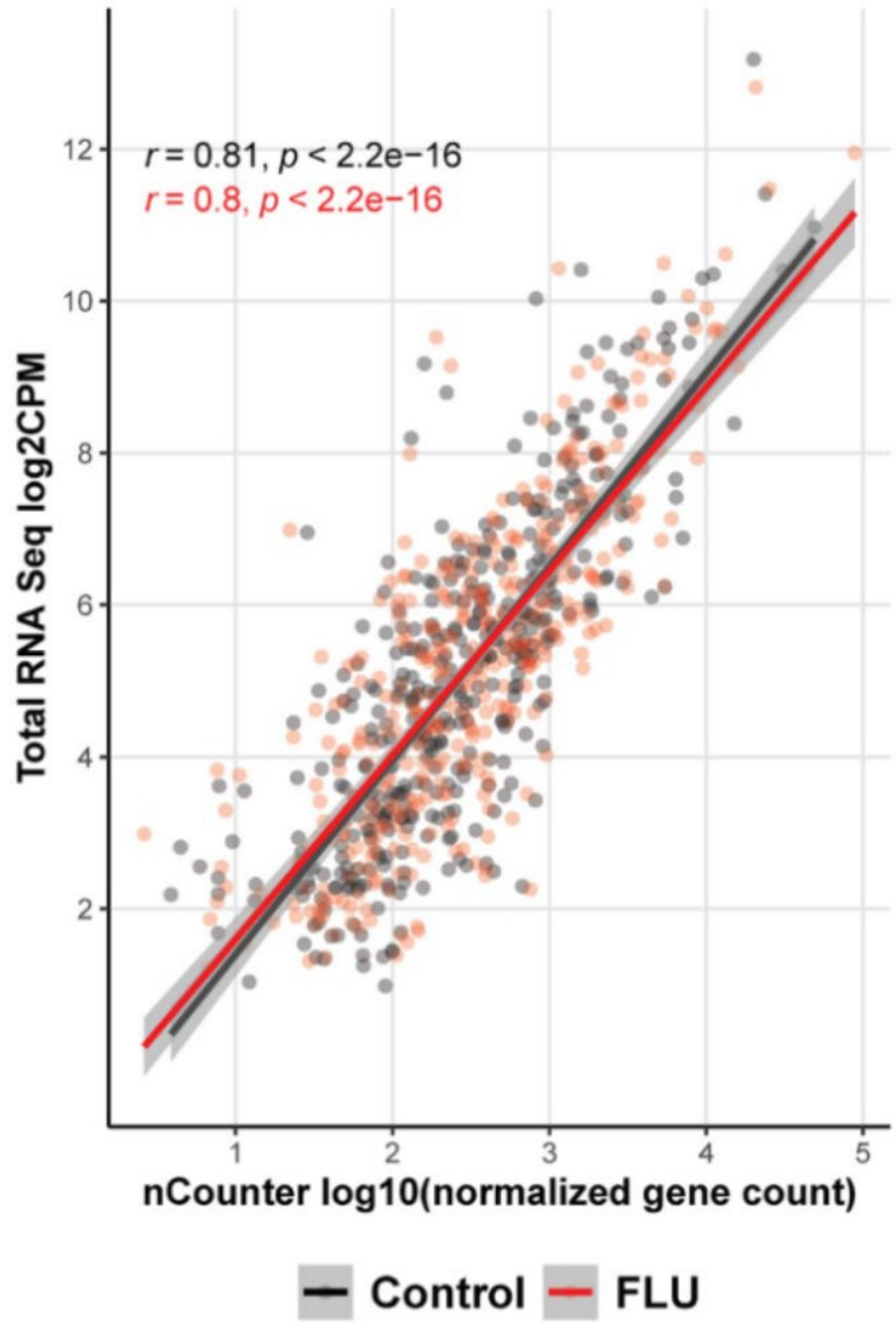

This dot plot shows correlations between placental log2-transformed RNA-Seq counts per million and log10-transformed Nanostring nCounter counts. Each point is the mean expression of a gene present in both datasets from FLU (red, N=405) or CTRL (black, N=405) samples. Pearson r and p-values are shown, with the best-fit line and standard error.

Figure S8. Cluster 1 Differentially Expressed Genes in Figure 4C

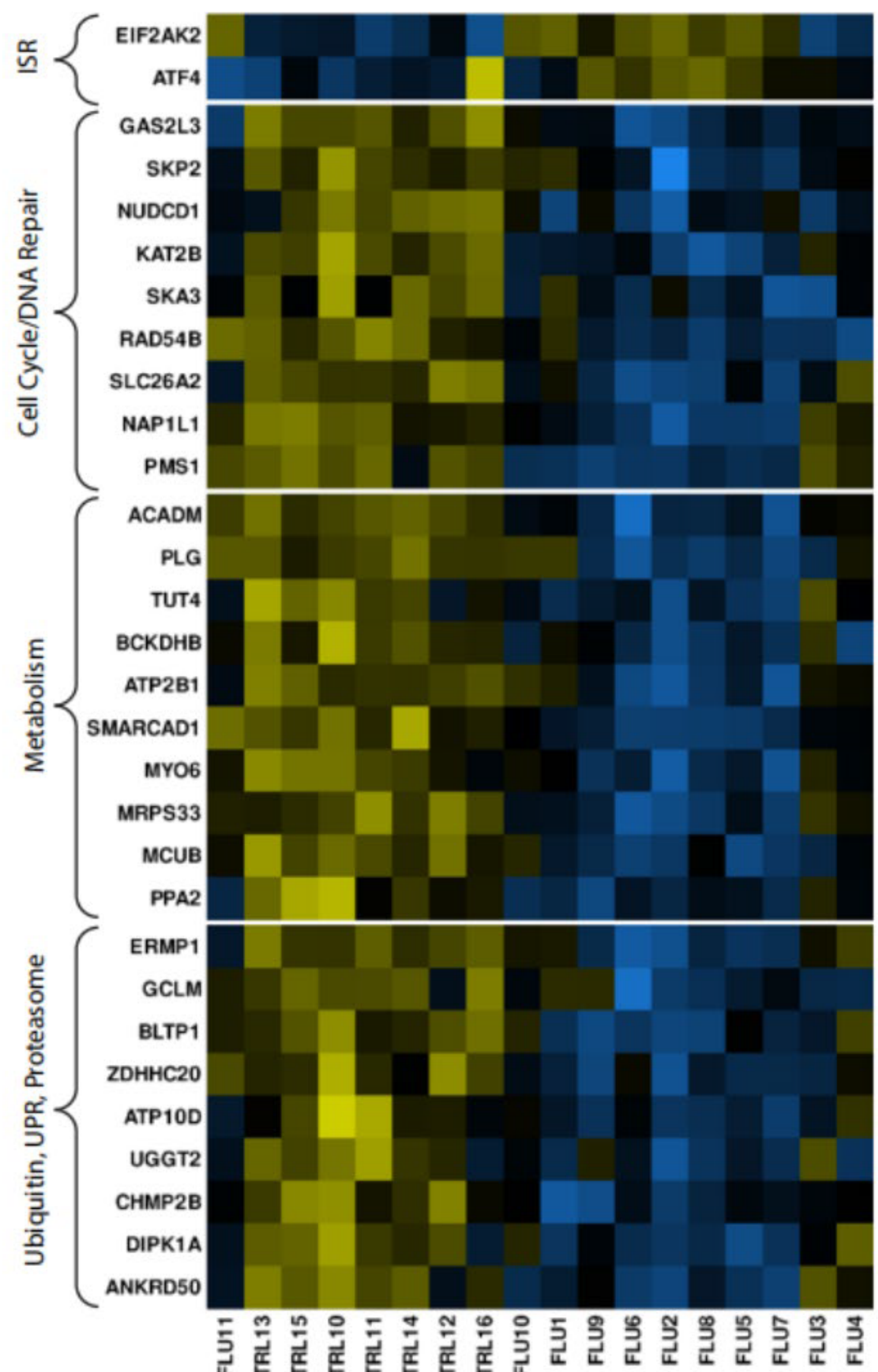

This is a heatmap showing a select set of differentially expressed genes found in Cluster 1 from the Figure 4C heatmap. The data represents normalized counts from the placental total RNA-Seq data from the FLU (N=11) and CTRL (N=7) cohorts. Each column represents an individual placental sample, and each row represents an individual gene. The yellow-blue color intensity indicates the gene z-score which was calculated based on the mean expression level of all animals for each gene. These genes have functions impacted by the viral-associated ISR, such as cell cycle/DNA repair, metabolism, ubiquitination, unfolded protein response (UPR), and proteasome activity. The viral-associated ISR activator (*EIF2AK2*) and mediator (*ATF4*) are shown at the top.

Figure S9. Cluster 2 Differentially Expressed Genes in Figure 4C

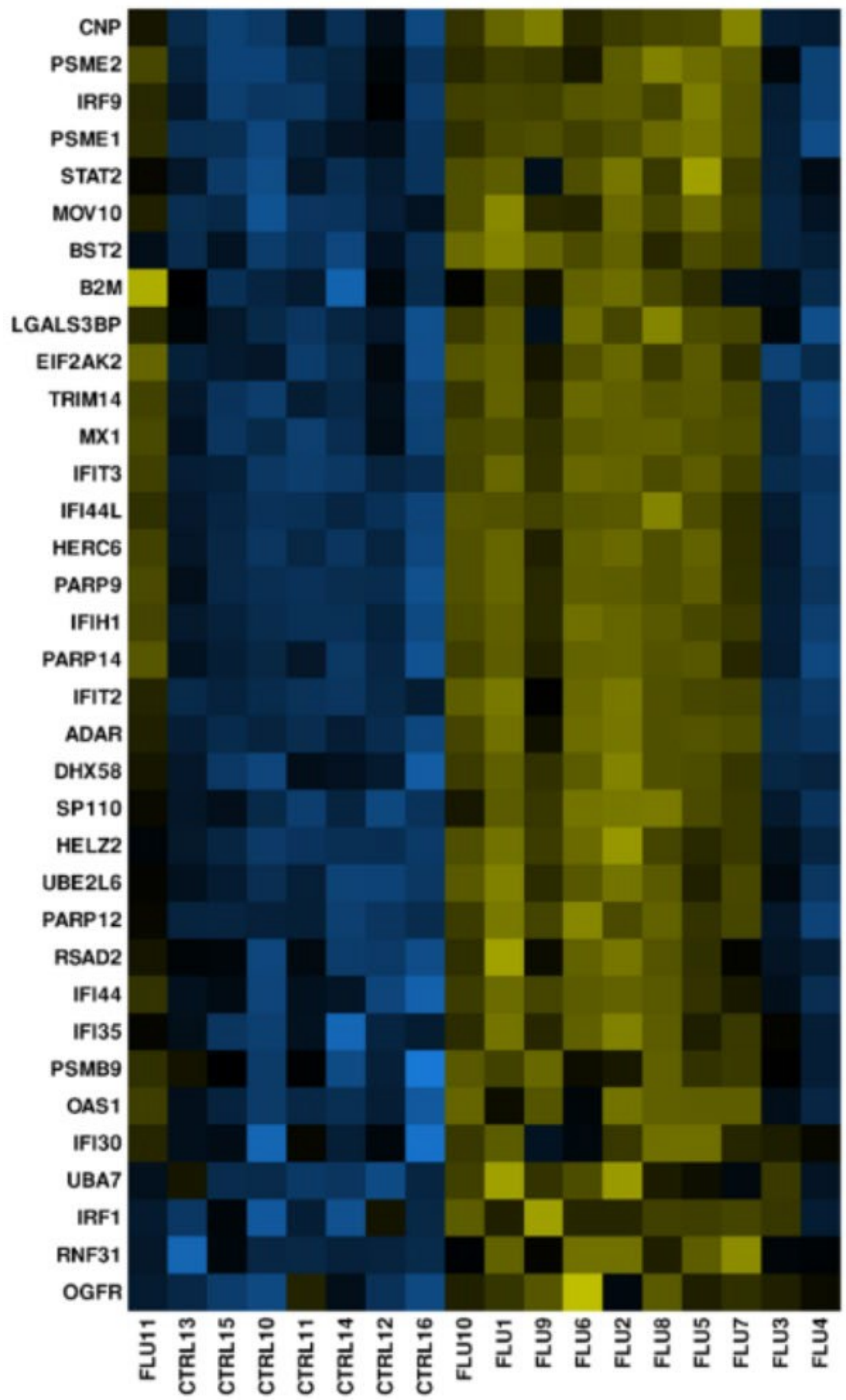

This is a heatmap showing a set of differentially expressed genes found in Cluster 2 from the Figure 4C heatmap. The data represents normalized counts from the placental total RNA-Seq data from the FLU (N=11) and CTRL (N=7) cohorts. Each column represents an individual placental sample, and each row represents an individual gene. The yellow-blue color intensity indicates the gene z-score which was calculated based on the mean expression level of all animals for each gene. An over-representation analysis (ORA) was performed on cluster 2 genes from panel A. Rows are genes in the Molecular Signatures Database Gene Set: Hallmark Interferon Alpha Response.

Figure S10. Over-Representation Analysis of Cluster 2 Differentially Expressed Genes in Figure 4C

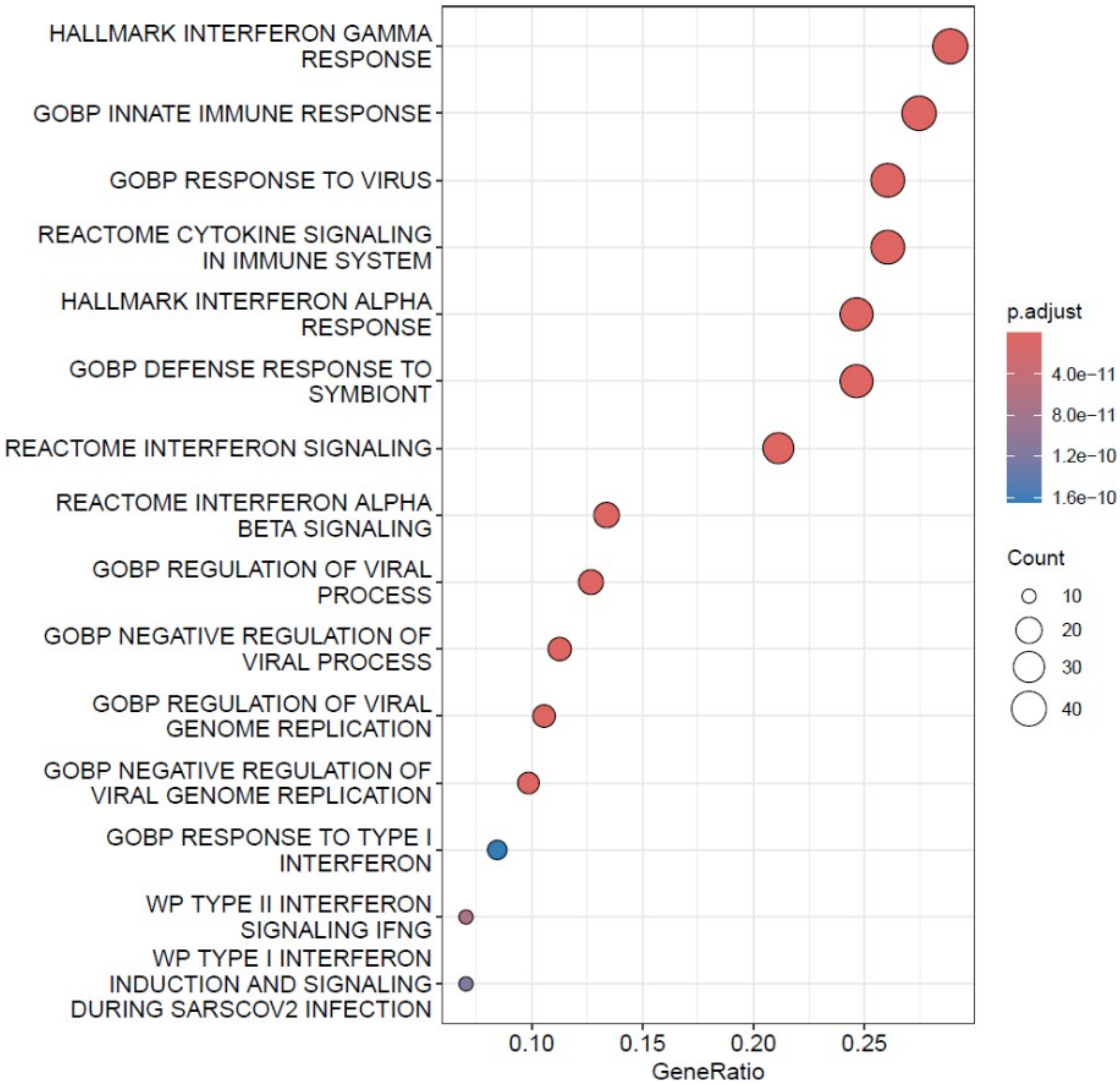

This is a bubble plot showing significantly enriched pathways from gene set over-representation analysis. Each row represents a pathway from either Gene Ontology Biological Process (GOBP), Reactome, Hallmark, or WikiPathways databases. The x-axis indicates the gene ratio (proportion of input genes associated with each pathway), while bubble size reflects the number of genes contributing to the enrichment. Bubble color corresponds to adjusted p values, with warmer colors denoting higher statistical significance. Enriched pathways include strong signatures of Type I and type II interferon signaling, viral genome regulation, and antiviral defense, highlighting the robust activation of immune responses.

Figure S11. Correlations Between Placental Cytokines and Maternal Disease Indicators

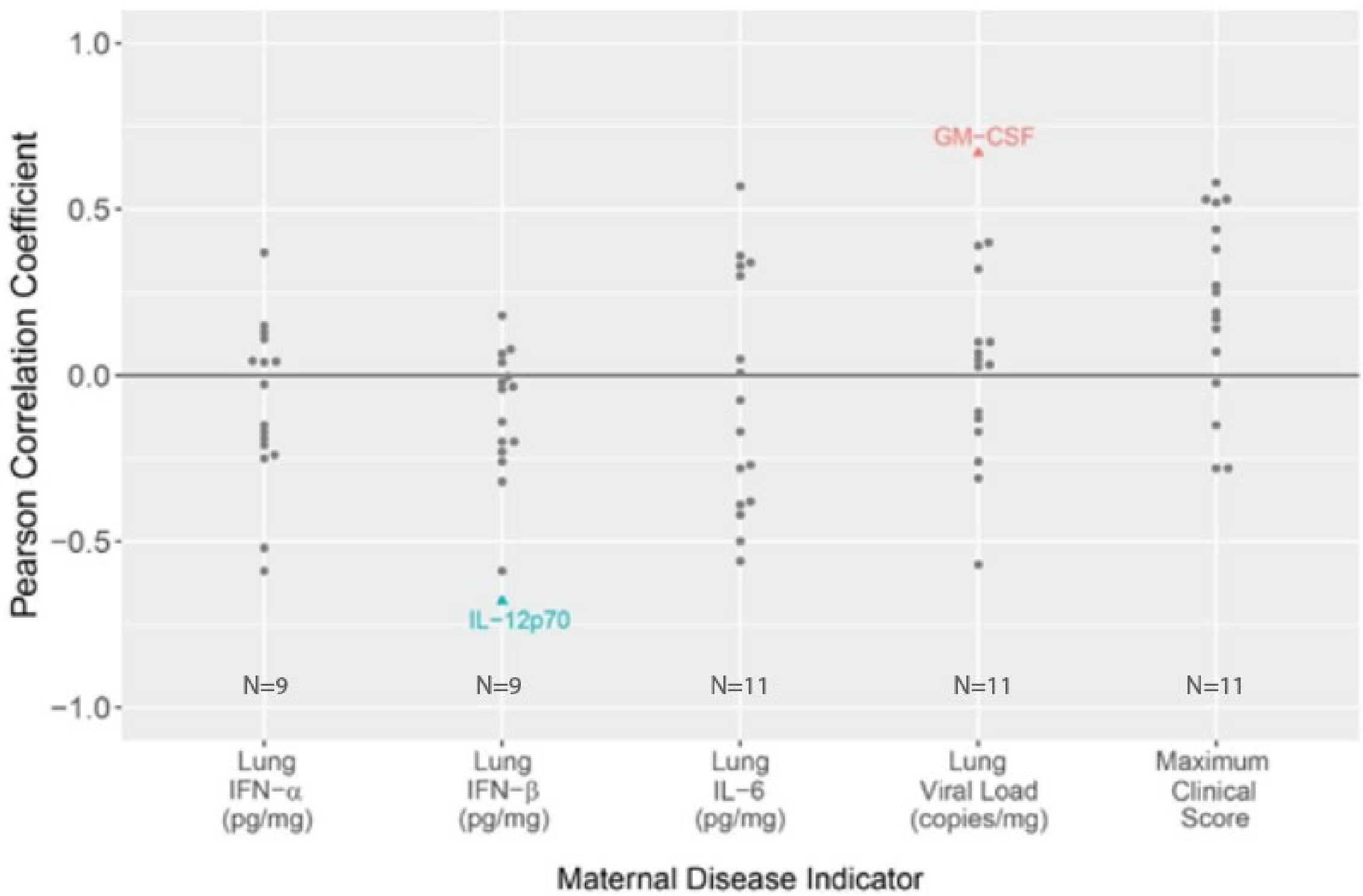

Each point represents a Pearson correlation analysis that was performed between placental cytokine concentrations with metrics of maternal IAV disease: lung IFN-alpha concentration, lung IFN-beta concentration, lung IL-6 concentration, maximum clinical score, and lung viral load. Cytokine concentrations and viral load values were log10-transformed before running the correlation. The correlation coefficient, R, was plotted. Correlations with a p-value less than 0.5 are shown with a colored triangle, and correlations with a p-value greater than 0.5 are shown with a black dot. The names of cytokines with significant correlations are indicated. The N is shown at the bottom of each column.

Figure S12. Correlations Between Placental Immunophenotyping and Maternal Disease Indicators

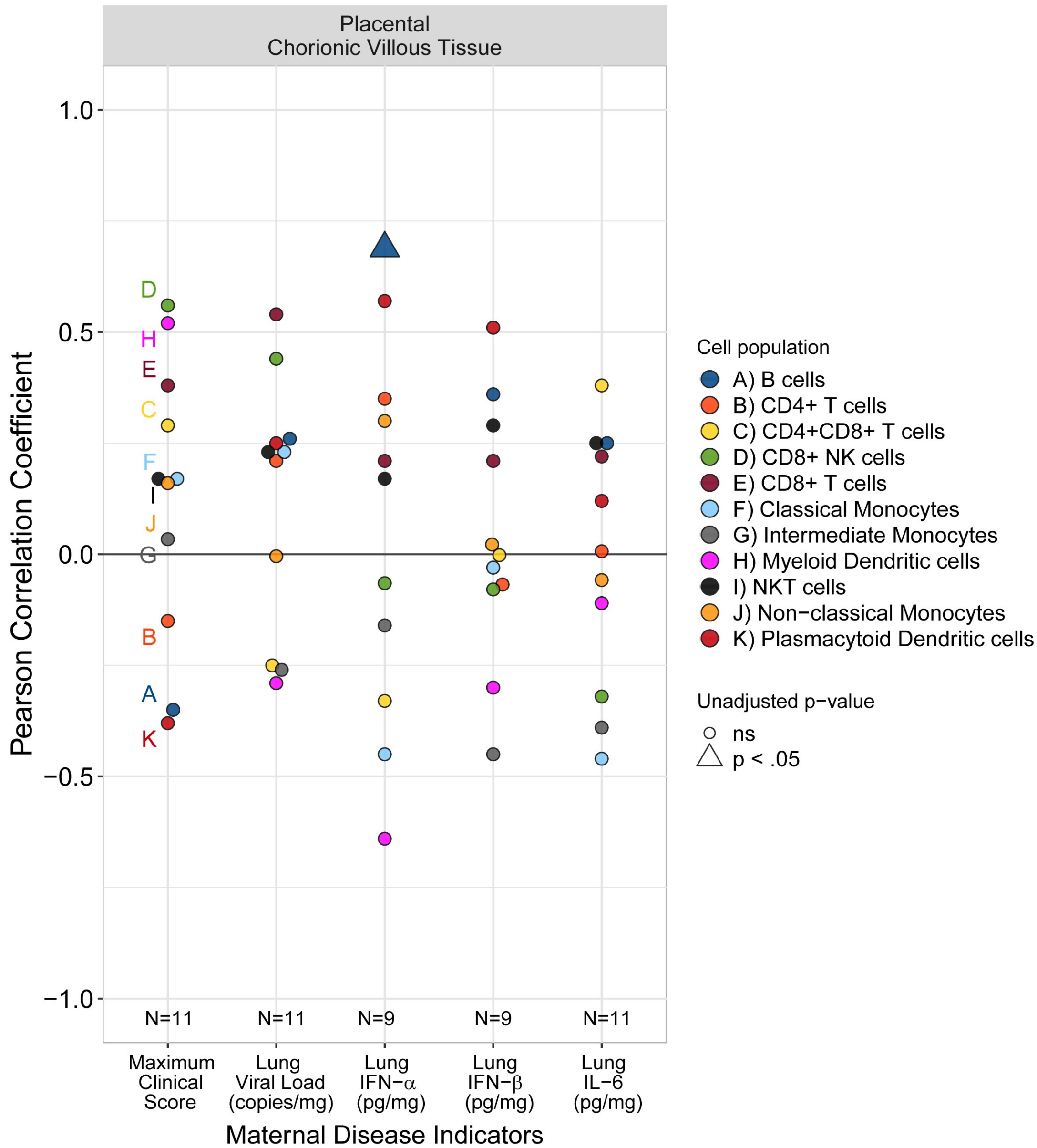

Pearson correlation analysis between maternal disease indicators (maximum clinical score, viral load, maternal lung IFN-α, IFN-β, and IL-6) and placental immune cell populations. The Pearson correlation coefficient is shown on the y-axis, and the maternal disease indicator is shown on the x-axis. Each immune cell population is shown by color and letter to aid in distinguishing populations from one another. A line connects each Pearson correlation coefficient from the same immune cell population, allowing tracking of similarities in positive and negative coefficients. Triangles show significant unadjusted p-values. The N is shown at the bottom of each column.

Figure S13. Gestational Age Subsetting of Fetal Immunophenotyping

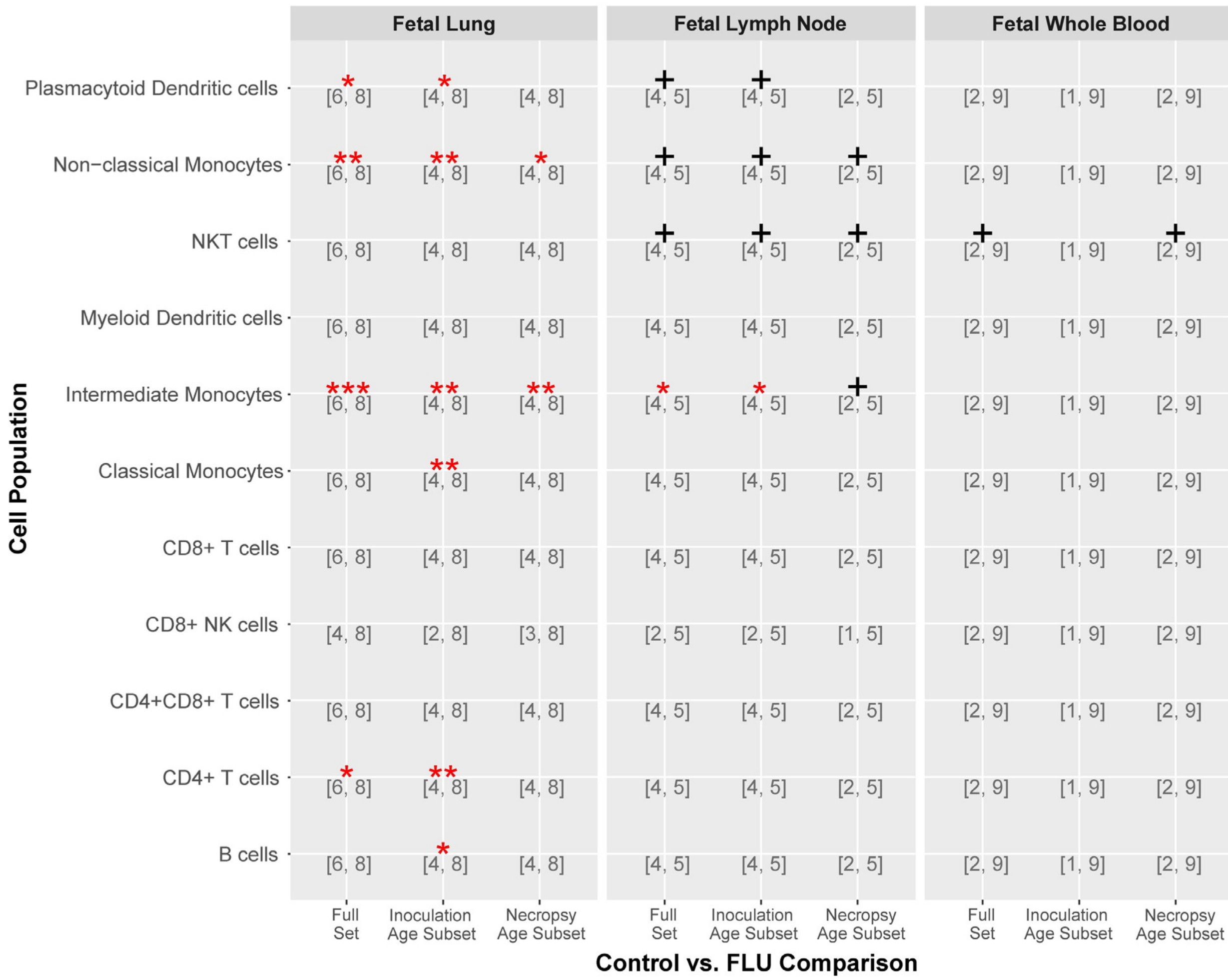

This figure illustrates the significance of the comparisons between CTRL versus FLU animals for a given cell population (y-axis) in the fetal lung (left panel), fetal lymph node (middle panel), and fetal whole blood (right panel). The method of subsetting is indicated on the x-axis and derives from Table S4. The full set includes all CTRL and FLU animals for the comparison. The p-values for each comparison were generated using a Wilcoxon rank-sum test and indicated with a plus sign if $0.05<p<0.1$ or red star(s) if significant (plus sign: $0.1 < p \leq 0.05$; 1 star: $p<0.05$; 2 stars: $p<0.01$, 3 stars: $p<0.001$). The sample size for each comparison is shown in brackets, with the left number indicating the CTRL sample size and the right number indicating the FLU sample size. 'NA' is shown to indicate where the cohort sample size was not sufficient to run the comparison.

Figure S14. Immunophenotyping of the Fetal Lung

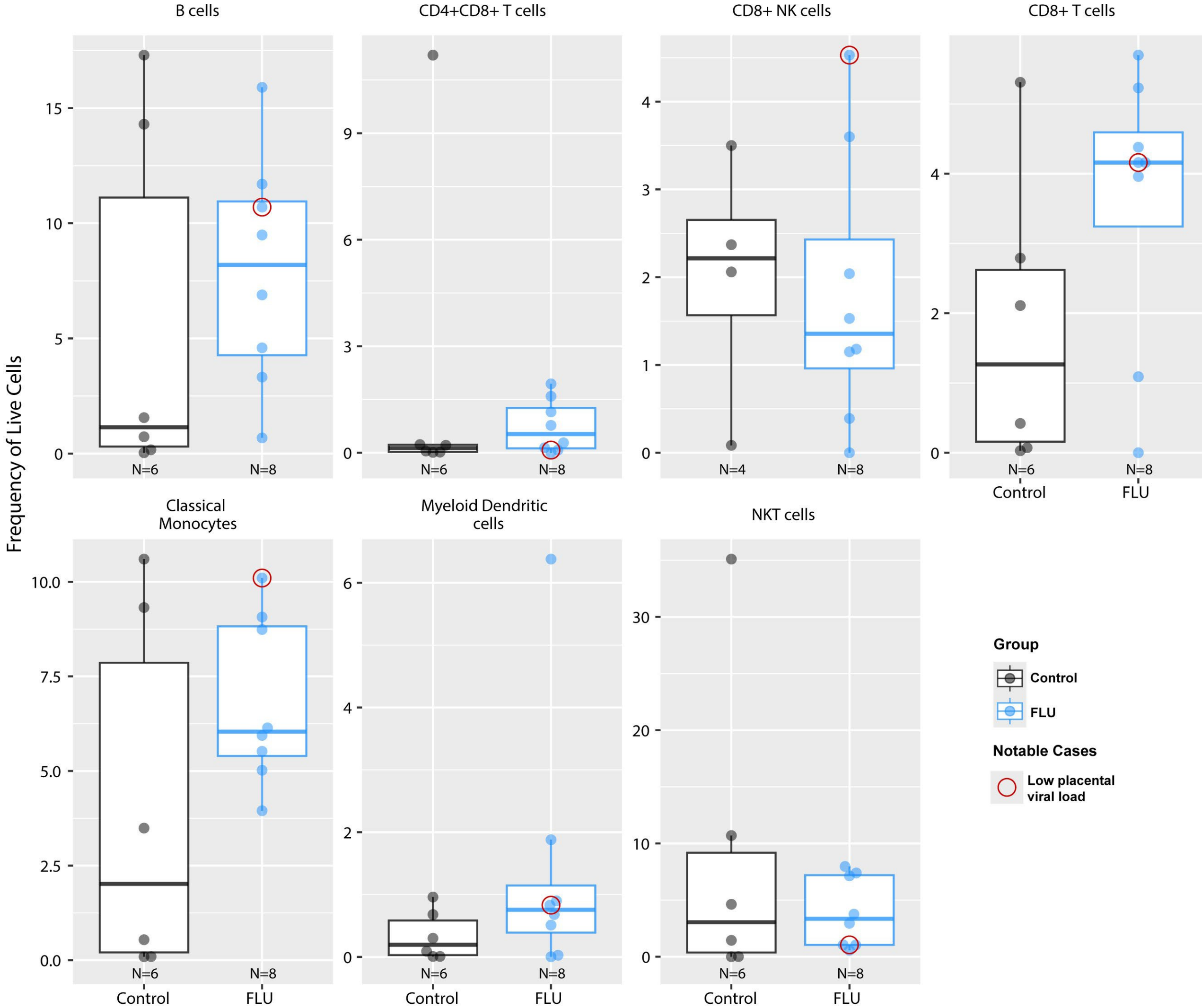

This figure illustrates the frequency of live cell populations (y-axis) of immune cell populations within the fetal lung, as determined by immunophenotyping (flow cytometry). The red circle indicates the frequency of live cells in the fetal lung from the pregnancy with a low IAV viral load in the placenta. Immunophenotyping was not done on fetal tissues from stillbirth. The N is shown at the bottom of each panel. Abbreviations: NKT, natural killer T cells.

Figure S15. Immunophenotyping of the Fetal Lymph Node

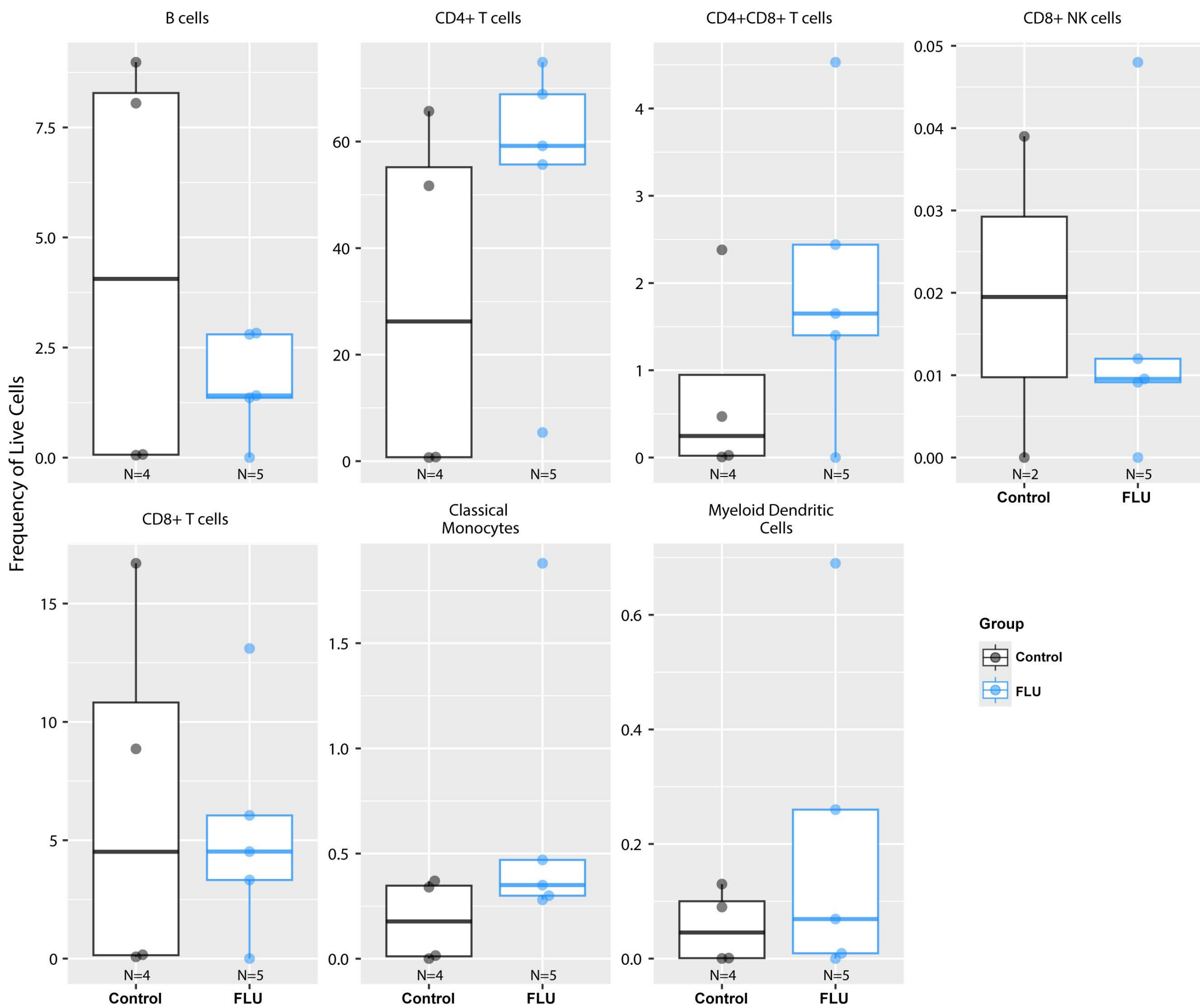

This figure illustrates the frequency of live cell populations (y-axis) of immune cell populations within the fetal mesenteric lymph nodes, as determined by immunophenotyping (flow cytometry). The red circle indicates the frequency of live cells in the fetal lung from the pregnancy with a low IAV viral load in the placenta. Immunophenotyping was not done on fetal tissues from stillbirth. The N is shown at the bottom of each panel.

Figure S16. Immunophenotyping of Fetal Whole Blood

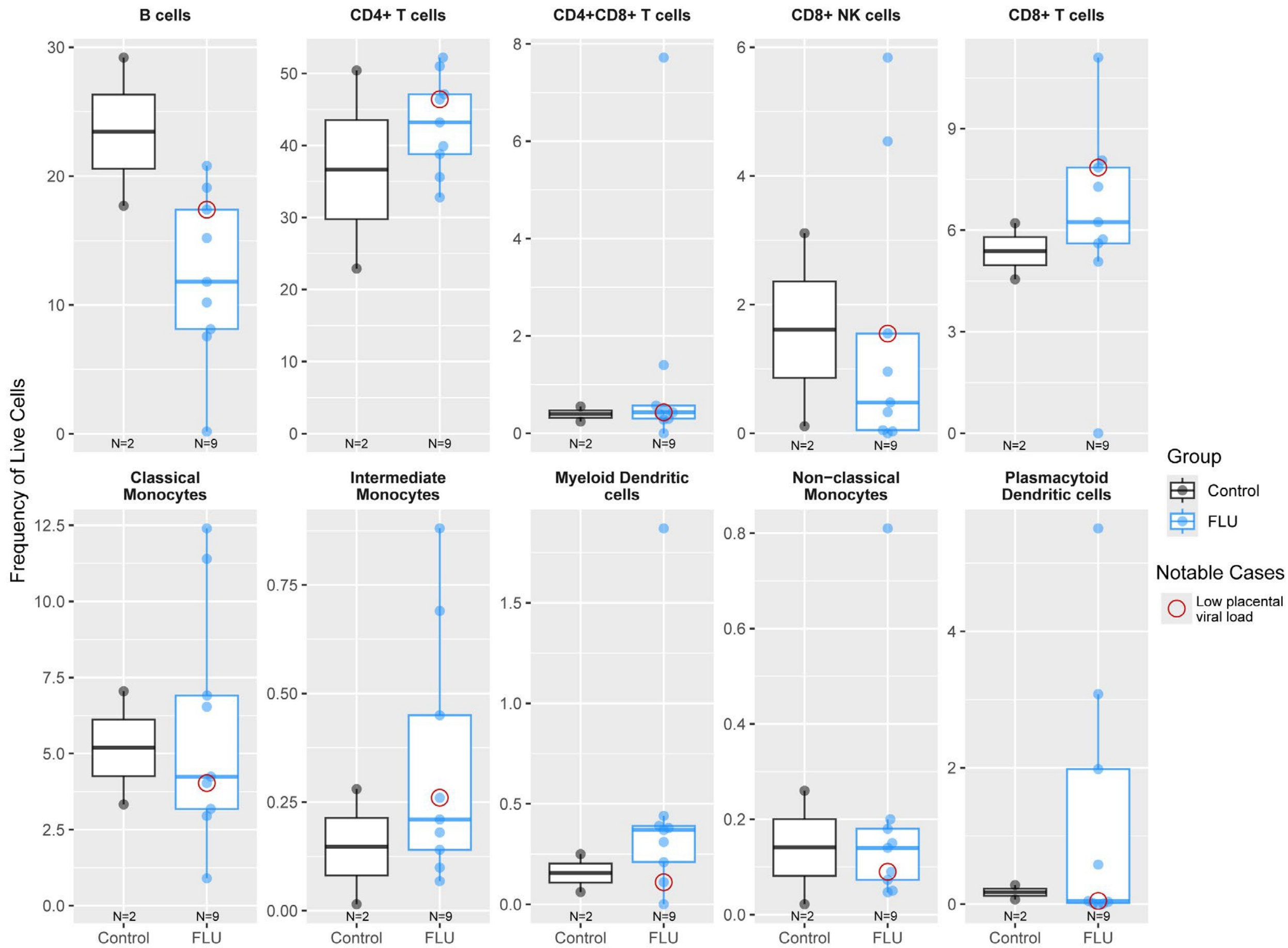

This figure illustrates the frequency of live cell populations (y-axis) of immune cell populations within the fetal whole blood, as determined by immunophenotyping (flow cytometry). The red circle indicates the frequency of live cells in the fetal lung from the pregnancy with a low IAV viral load in the placenta. Immunophenotyping was not done on fetal tissues from stillbirth. The N is shown at the bottom of each panel.

Figure S17. Pearson Correlation Coefficients Between Maternal Lung Disease Indicators and Fetal Immune Cell Populations

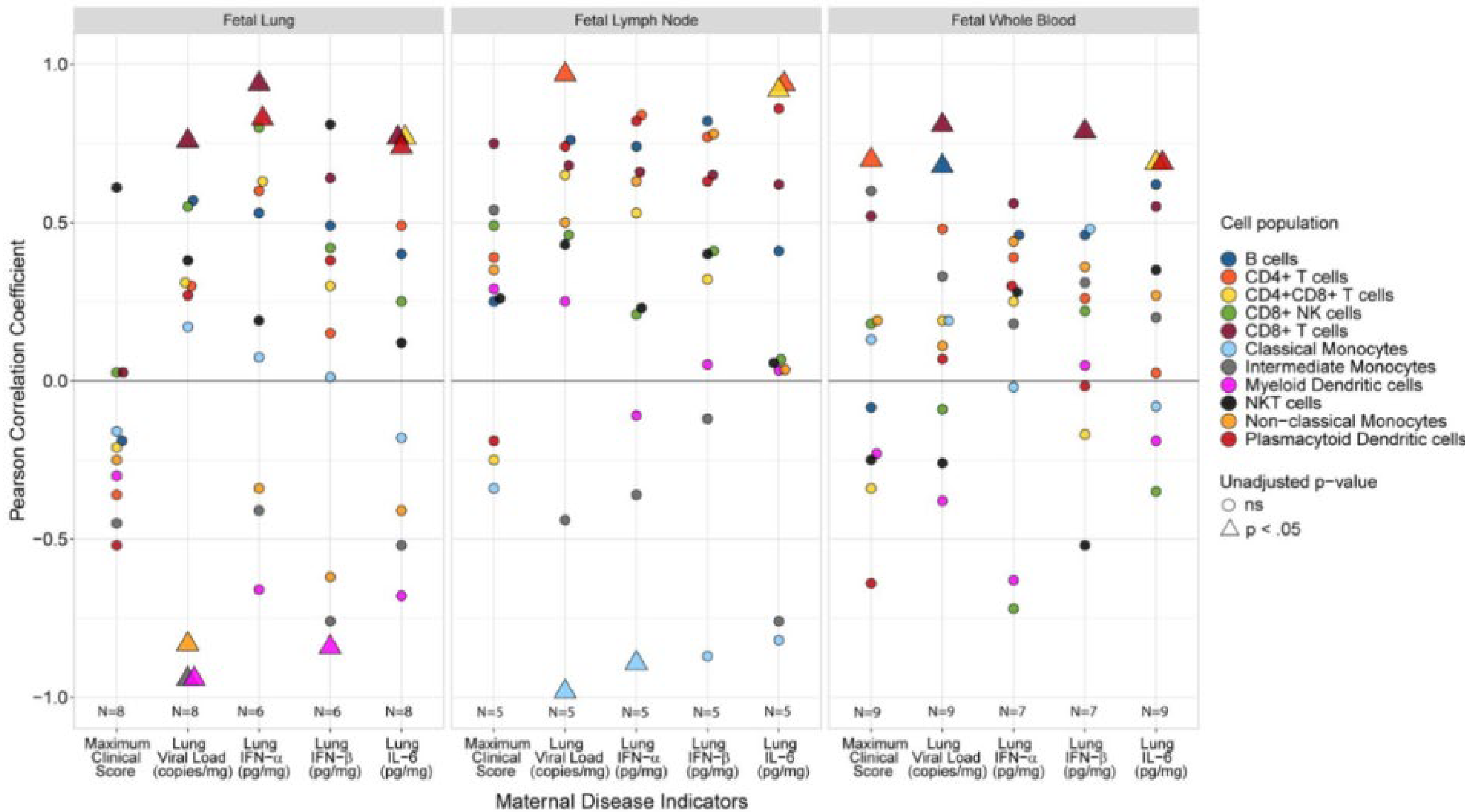

Pearson correlation analysis between maternal disease indicators (maximum clinical score, lung viral load, lung IFN-a/IFN-b/IL-6 concentration) and fetal immune cell populations in the lungs, lymph nodes, and whole blood of IAV-infected animals. The Pearson correlation coefficient is shown on the y-axis, and the maternal disease indicator is shown on the x-axis. Each immune cell population is indicated by a given color. Each dot/triangle represents a correlation that was run between a maternal disease indicator (specified by the column) and a cell population (specified by the color) using FLU animals. The number of FLU animals used to run each correlation is consistent for each column, and the N is shown at the bottom of the column. A total of 55 unique correlations were run for each tissue type. Significant correlations (unadjusted p<0.5) are shown as triangles, and non-significant correlations (unadjusted p≥0.5) are shown as dots.

Figure S18. Representative Gating Strategy for Granulocytes Immunophenotyping

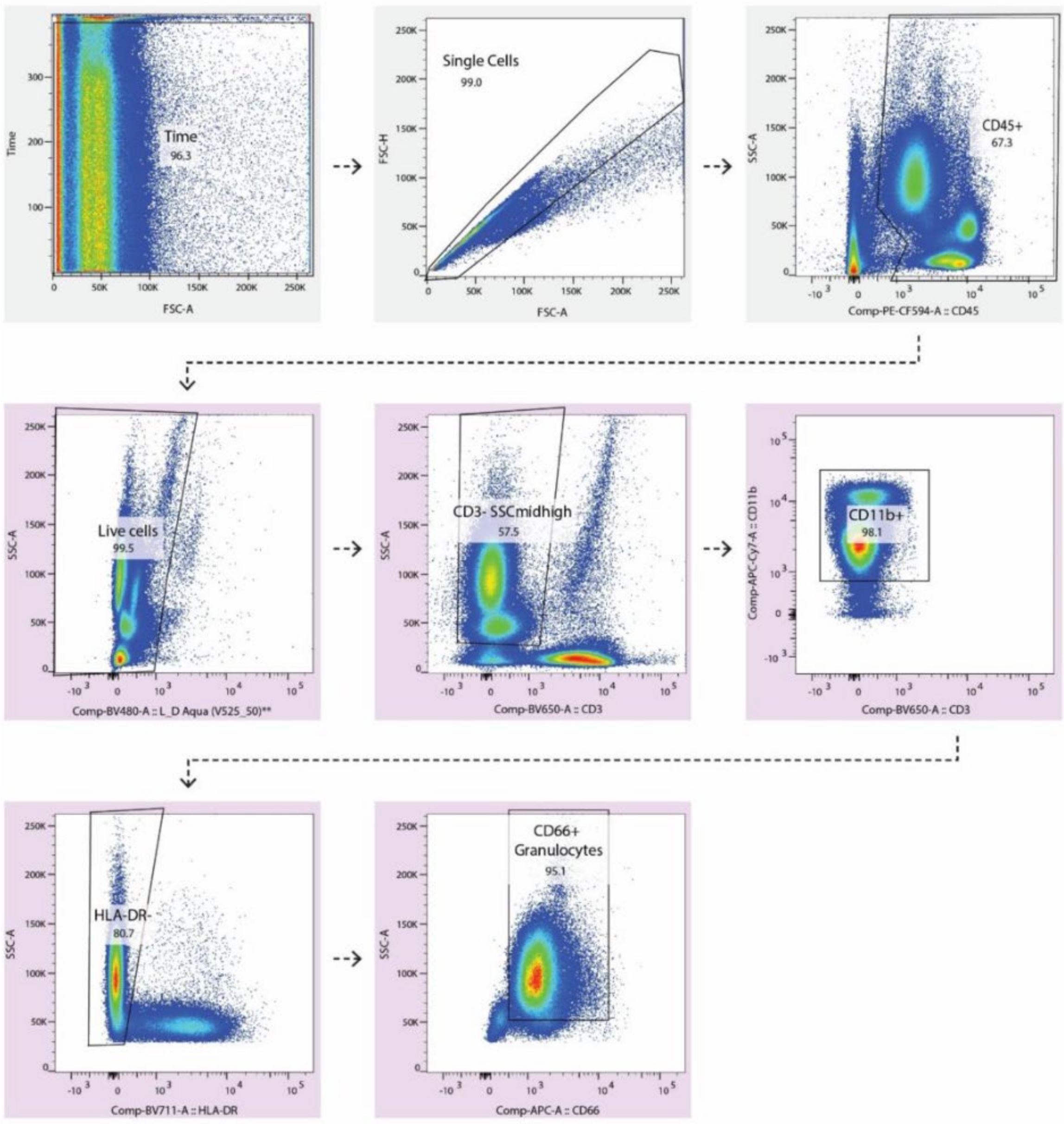

Single cells were analyzed by multiparametric flow cytometry. Doublets were excluded by forward scatter height (FSC-H) versus forward scatter area (FSC-A) gating. Leukocytes were gated by side scatter (SSC-A) and CD45 to distinguish granulocytes from non-hematopoietic events. Within CD45+ cells, viable cells were identified by exclusion of the viability dye (Aqua). Next, cells were identified as either CD3 negative, mid- and high-SSC-A cells. CD66+ granulocytes were further discriminated by gating to isolate CD11b+, HLADR-, and CD66+ cells. Shown is a representative gating scheme from one subject. The parent population name is overlayed on the plot in large text.

Figure S19. Representative Gating Strategy for Immunophenotyping of Lymphoid Cells

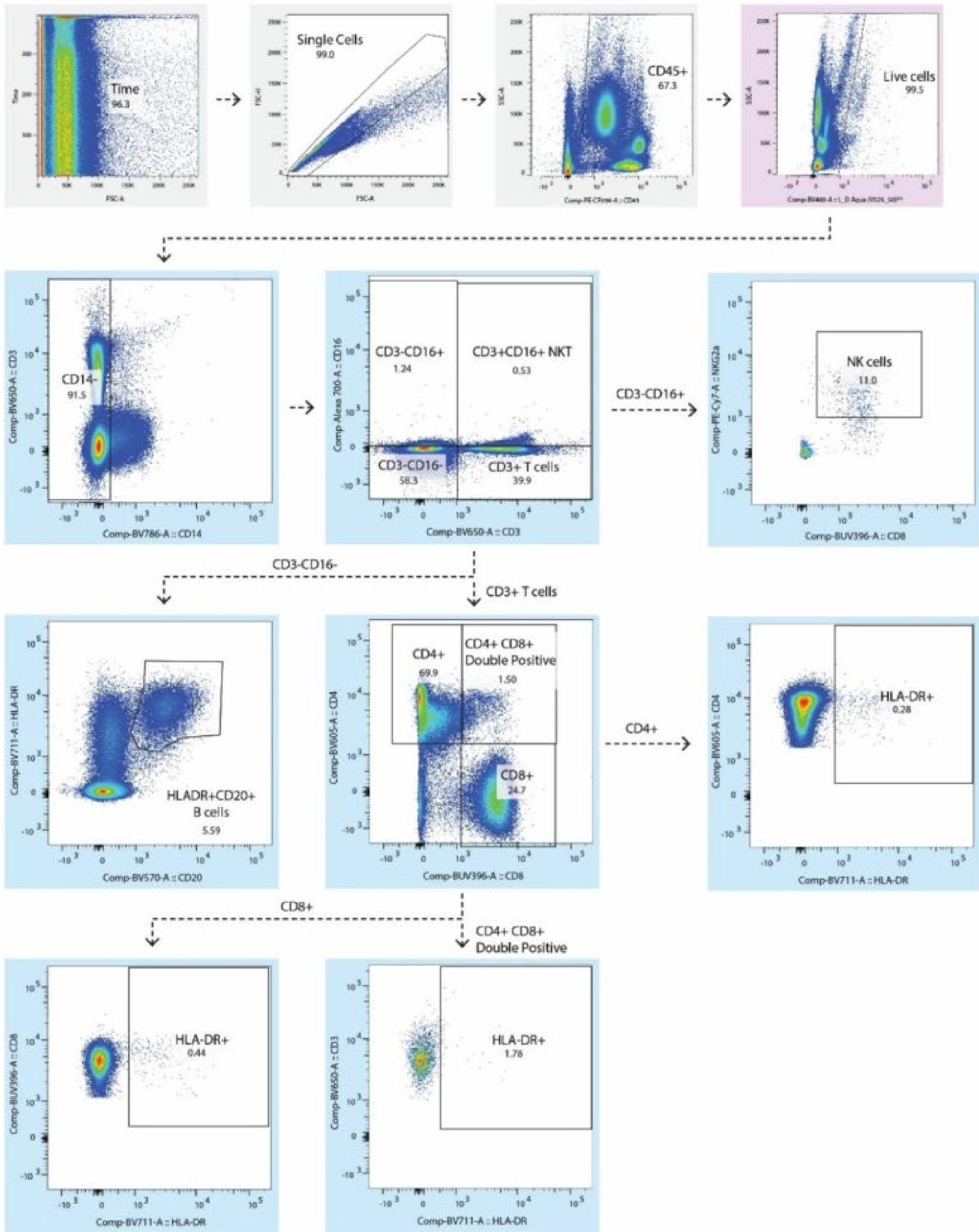

Single cells were analyzed by multiparametric flow cytometry. Doublets were excluded by forward scatter height (FSC-H) versus forward scatter area (FSC-A). Leukocytes were gated by side scatter (SSC-A) and CD45 to distinguish granulocytes from non-hematopoietic events. Within CD45+ cells, viable cells were identified by exclusion of the viability dye (Aqua). Within the lymphocyte gate, major lineages were defined using CD3, CD16, HLA-DR, and CD20. Natural

killer (NK) cells were identified as CD3-/CD16+. B cells were defined as CD3-/CD16-/HLA-DR+CD20+. CD3+ T cells were subdivided into CD4+, CD8+, and double-positive (CD4+CD8+) populations. Activated T cell subsets were further characterized based on HLA-DR expression, including CD4+HLA-DR+ T cells, CD8+HLA-DR+ T cells, and CD4+CD8+HLA-DR+ T cells. Shown is a representative gating scheme from one subject. The parent population name is overlaid on the plot or next to its corresponding arrow in large text.

Figure S20. Representative Gating Strategy for Monocyte Immunophenotyping

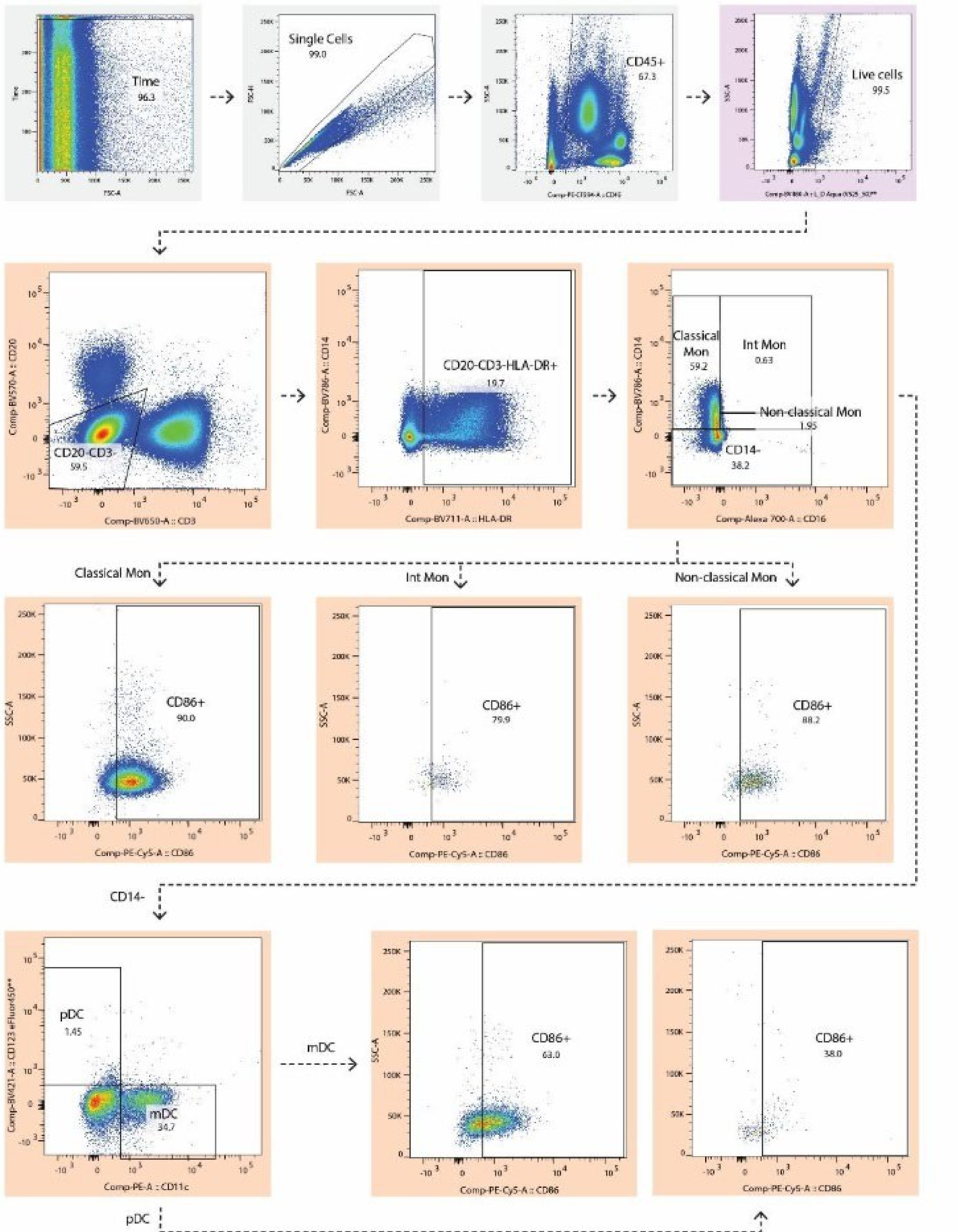

Single cells were analyzed by multiparametric flow cytometry. Doublets were excluded by forward scatter height (FSC-H) versus forward scatter area (FSC-A). Leukocytes were gated by side scatter (SSC-A) and CD45 to distinguish granulocytes from non-hematopoietic events. Within CD45+ cells, viable cells were identified by exclusion of the viability dye (Aqua). Monocytes and dendritic cells were gated from CD45+ leukocytes to select CD20-CD3- cells. (D) Within the monocyte population, subsets were defined using CD14, CD16, and CD86 expression: classical monocytes (CD14++CD16-CD86+), intermediate monocytes (CD14+CD16+CD86+), and non-

classical monocytes (CD14^{lo}CD16++CD86+). (E) Dendritic cells were identified by exclusion of lineage markers and further subdivided based on CD11c, CD123, and CD86 expression: myeloid dendritic cells (CD11c+CD123-CD86+) and plasmacytoid dendritic cells (CD11c-CD123+CD86+). Shown is a representative gating scheme from one subject. The parent population name is overlaid on the plot or next to its corresponding arrow in large text.
